# Tracking Neural Progenitor Cell Migration in the Rodent Brain Using Magnetic Resonance Imaging

**DOI:** 10.3389/fnins.2018.00995

**Published:** 2019-01-11

**Authors:** Christiane L. Mallett, Dorela D. Shuboni-Mulligan, Erik M. Shapiro

**Affiliations:** ^1^Molecular and Cellular Imaging Laboratory, Department of Radiology, Michigan State University, East Lansing, MI, United States; ^2^Institute for Quantitative Health Science and Engineering, Michigan State University, East Lansing, MI, United States

**Keywords:** MRI – magnetic resonance imaging, neural progenitor and stem cells, cell transplantation, neurogenesis, rodents (rats, mice, guinea pigs, voles)

## Abstract

The study of neurogenesis and neural progenitor cells (NPCs) is important across the biomedical spectrum, from learning about normal brain development and studying disease to engineering new strategies in regenerative medicine. In adult mammals, NPCs proliferate in two main areas of the brain, the subventricular zone (SVZ) and the subgranular zone, and continue to migrate even after neurogenesis has ceased within the rest of the brain. In healthy animals, NPCs migrate along the rostral migratory stream (RMS) from the SVZ to the olfactory bulb, and in diseased animals, NPCs migrate toward lesions such as stroke and tumors. Here we review how MRI-based cell tracking using iron oxide particles can be used to monitor and quantify NPC migration in the intact rodent brain, in a serial and relatively non-invasive fashion. NPCs can either be labeled directly *in situ* by injecting particles into the lateral ventricle or RMS, where NPCs can take up particles, or cells can be harvested and labeled *in vitro*, then injected into the brain. For *in situ* labeling experiments, the particle type, injection site, and image analysis methods have been optimized and cell migration toward stroke and multiple sclerosis lesions has been investigated. Delivery of labeled exogenous NPCs has allowed imaging of cell migration toward more sites of neuropathology, which may enable new diagnostic and therapeutic opportunities for as-of-yet untreatable neurological diseases.

## Introduction

The potential of using stem cells to repair the brain after traumatic injury (Lepore et al., 2006; Lin et al., 2018; Mundim et al., 2019), stroke (Hoehn et al., 2002; Zhang et al., 2003; Huang et al., 2014), cancer (Zhang et al., 2004; Muldoon et al., 2006; Barish et al., 2017), Parkinson’s disease (Lamm et al., 2014; Ramos-Gomez and Martinez-Serrano, 2016), neonatal hypoxia-ischemia (Obenaus et al., 2011) and multiple sclerosis (Ben-Hur et al., 2007; Reekmans et al., 2011; Harris et al., 2018), as well as other diseases, is significant. For example, when NPCs are injected directly into the brain in rodent models of ischemic stroke, there are marked improvements in both behavioral and physiological markers (Obenaus et al., 2011; Daadi et al., 2013; Huang et al., 2014). NPC independently migrate through tissue to the site of disease using a chemotaxis sense, moving toward the presence of cytokines caused by disease, a process called homing (Peng et al., 2004; Kokovay et al., 2010; Reaux-Le Goazigo et al., 2013). In glioma, NPCs actively migrate to tumors (Aboody et al., 2000; Diaz-Coranguez et al., 2013; Bago et al., 2017) and the mere presence of NPCs could impede the growth and proliferation of tumors (Glass et al., 2005). The effectiveness of NPCs as therapeutics may be due to integration of cells into existing circuitry, or through their effect on the damaged brain tissue through the secretion of factors or extracellular vesicles (e.g., exosomes) that influence inflammation, neovascularization, and plasticity (Boese et al., 2018).

The SVZ is one of two well-characterized regions of the brain where neurogenesis persists after development is complete, the other being the subgranular zone of the dentate gyrus (Gage, 2002; Gonzalez-Perez, 2012). NPCs originating in the SVZ migrate to the OB in a well-organized chain of cells called the RMS.

**FIGURE 1:**
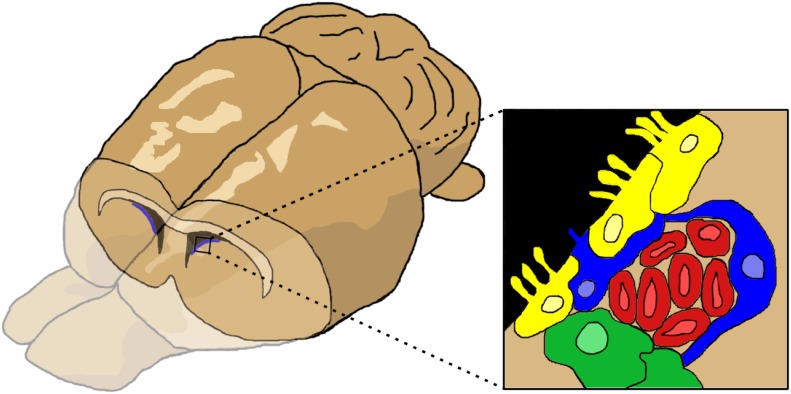
The cells of the subventricular zone (SVZ). The SVZ is located just below the lateral ventricles. Cells (inset) consist of ependymal cells (yellow), type B or astrocytes (blue), type C or transit amplifying cells (green), and type A cells or neuroblasts (red). Figure inspired by García-Verdugo et al. (1998), Ihrie and Álvarez-Buylla (2011).

The SVZ is located proximal to the lateral ventricles (Figure 1). The region is populated by several cell types. The ependymal cells and type B cells (astrocytes) line the ventricle wall. Astrocytes are divided into two subtypes: type B1 cells have apical projections into the cerebral spinal fluid (CSF) while type B2 cells do not contact the ventricle. Type B cells can differentiate into type C cells (transit amplifying cells or intermediate precursor cells), which then differentiate into type A cells (neuroblasts). Neuroblasts migrate through a sheath formed by type B cells toward the OB, forming the RMS. In the developing rodent brain, a constant stream of regenerating NPCs travel from the SVZ through the RMS to the OB, where they differentiate and replace dead neurons or create/reinforce neural pathways (reviewed in Ihrie and Álvarez-Buylla, 2011; Butti et al., 2014). NPC proliferation and migration can be upregulated and redirected by signals along inflammatory signaling pathways or by growth and neurotrophic factors (reviewed in Christie and Turnley, 2013). Of note, the SVZ has a mix of NSCs (multipotent, self-renewing) and NPCs (pluripotent, limited self-renewal), but the exact proportion of each cell type in the SVZ and their relevant markers remain in dispute (Seaberg and van der Kooy, 2003; Abrous et al., 2005). Although these characteristics are important, for simplicity we will use the general term, NPC, to refer to all cells in brain with self-renewing potential.

Cell migration in the RMS has been studied on excised tissues using a variety of methods to label cells. One *in situ* method is to inject viral vectors into the SVZ or the lateral ventricle leading to transfection of nearby cells; this has been used to transfer genes encoding for fluorescent (Suzuki and Goldman, 2003; Rogelius et al., 2005; Ventura and Goldman, 2007) or bioluminescent proteins (Guglielmetti et al., 2014). Such injections can also be used to label cells with BrdU, which incorporates into the DNA of dividing cells and can then be detected using histologic techniques (Betarbet et al., 1996; Arvidsson et al., 2002; Mundim et al., 2019). Each of these methods shares the drawback that analysis can only be performed after excision of the tissue after the animal has been euthanized, such that only a single time point per animal can be assessed, and this is usually done on histological sections that further limit the study by reducing the sample size. Migration of fluorescent cells can be detected using two-photon microscopy through a cranial window (e.g., Lin et al., 2018). Using this method, only a limited area of the brain can be imaged. Bioluminescence imaging can also be used to track transplanted cells, but has limited resolution (e.g., Rogall et al., 2018).

Studying NPCs *in vivo* using magnetic resonance imaging (MRI) avoids some of these drawbacks but can introduce new challenges. In this technique, cells are labeled with superparamagnetic iron oxide particles (SPIO), either *in vitro* or *in situ*, and the animal can be serially imaged, allowing for tracking of NPCs and monitoring of treatment response. The SPIO cause local in homogeneities in the magnetic field that appear as hypointensities or dark contrast in gradient echo-based MR images. The artifact is much larger than the particle itself, to the extent that single labeled cells are detectable by MRI (Heyn et al., 2006a; Shapiro et al., 2006b), offering excellent sensitivity for tracking NPCs in the rodent brain. This technique has been used to track NPCs, immune cells, and cancer cells in the brain (Kleinschnitz et al., 2003; Heyn et al., 2006b; Shapiro et al., 2006a) and elsewhere in the body (Beckmann et al., 2003; Shapiro et al., 2006a; Tai et al., 2006). Compared to histological techniques, MRI allows for serial tracking within subjects, reducing the error variance associated with differences between subjects and allowing for a more complete understanding of a dynamic process through the power of repeated measures, without using ionizing radiation. MRI has the flexibility to provide both anatomical and functional data. For example, with MRI we can assess changes in lesion size for stroke (structure), then measure changes in perfusion and/or permeability (function) following NPC treatment (Jiang et al., 2005; Daadi et al., 2009, 2013). Unlike histology, MRI enables dynamic and repeated measures of these parameters over time and can sample the whole brain.

Our objectives in this review are to demonstrate the use of MRI to track NPCs in the rodent brain, describe the tools and methods of labeling cells, and discuss what we have learned about regeneration using imaging. Two main strategies are used for labeling NPCs: (1) injecting the iron oxide particles directly into the brain or ventricles to label proliferating NPCs *in situ*, or (2) labeling cells *in vitro* with iron oxide particles and then transplanting them into the animal either within the brain or vascular system. In both approaches, migration toward the OB or to the site of an injury can be monitored over time. As these techniques have matured, challenges related to the optimal way to label the cells, where the cells or particles should be injected, and how best to visualize and quantify the labeled cells have been defined by the many groups working on tracking NPCs *in vivo*. We will discuss the methods, current applications and future directions of both techniques.

## Iron Oxide Contrast Agents and Magnetic Cell Labeling

A variety of SPIO contrast agents are available for labeling cells *in vitro* and *in situ*. Micron-sized particles of iron oxide (MPIOs) are 0.86–1.63 μm diameter particles that can also be fluorescent. They have a high iron content (∼1 pg/particle), so that a single particle is detectable by MRI, but their polystyrene coating is non-biodegradable and is therefore not a clinically viable agent (Shapiro et al., 2004). Smaller dextran coated particles (SPIO) such as Feridex/Endorem (ferumoxides) and Resovist (ferucarbotran) have been shown to label NPC *in vitro* (Song et al., 2007; Lu et al., 2017) and are clinically approved, though as of this writing they are no longer available for purchase in North America. Feraheme (ferumoxytol), an ultrasmall iron oxide particle (USPIO) is clinically approved as a treatment for anemia and has been used in cell tracking studies, although not in NPCs transplantation in humans as of yet. Pre-clinically, these agents have been shown to effectively label human NSC *in vitro* and that labeled cells continue to home to disease in mice (Gutova et al., 2013). However, the FDA has recently issued a black-box warning because fatal allergic reactions were seen in some patients with anemia following intravenous administration of ferumoxytol. There are other dextran coated particles in development that are commercially (FeraTrack Direct; Aswendt et al., 2015; Kim et al., 2016) or laboratory (Song et al., 2007; Barrow et al., 2015) derived and have been applied to NSC tracking.

Iron oxide particles with unique features have been fabricated in individual laboratories and used for cellular imaging experiments. PLGA encapsulated iron oxide particles have been described as a clinically viable source of contrast for MRI-based cell tracking (Nkansah et al., 2011; Granot et al., 2014; Shapiro, 2015). These particles vary in size from 100 nm to 2 μm and efficiently package iron within their polymer shell comprised of a FDA-approved material. *In vitro* labeling of NPCs with these particles does not impair the ability of these cells to differentiate down neuronal, astrocyte or oligodendrocyte lineages (Granot et al., 2014). Magnetoliposomes consisting of SPIO enclosed in a phospholipid bilayer have been used to label NPCs *in situ* (Vreys et al., 2011), as well as custom-made targeted glyconanoparticles as described by Elvira et al. (2012).

Chemical tools that were originally developed for transfecting genes into cells have been adapted for cell labeling and can increase the efficiency of particle uptake into cells *in situ* or *in vitro*. These include the use of poly-L-lysine (PLL) (Frank et al., 2002; Daadi et al., 2009, 2013) or protamine sulfate (Guzman et al., 2008; Panizzo et al., 2009) which are co-incubated with particles and cells for *in vitro* labeling and co-injected with particles for *in situ* labeling. More complex methods for *in vitro* labeling include electroporation (Obenaus et al., 2011) or sonoporation (Xie et al., 2010) or a gene gun (Zhang et al., 2003; Jiang et al., 2005); all of these methods have been used with some success.

## Mri Protocols and Sequences

There are several methods for using MRI to detect magnetically labeled cells, with most manipulating some aspect of the local magnetic inhomogeneity caused by close proximity of the superparamagnetic nanoparticles. The most commonly reported methods make use of gradient recalled echo (GRE) based pulse sequences. The local magnetic inhomogeneity induced by the superparamagnetic nanoparticles accelerates the dephasing of water protons near the particles following radiofrequency excitation, essentially quenching the NMR signal. This results in a dark spot in the MRI image (Shapiro et al., 2006b). The sensitivity of this method to iron oxide nanoparticles can be increased by lengthening the echo time (TE), making the dark spot larger, but this can also result in image deformation with longer TE. Spin echo sequences can also be used to probe this phenomenon, but due to the ability to rapidly acquire 3D volumes using short flip angle and fast repetition time (TR), GRE techniques have advantages over spin echo techniques.

Another approach to exploit the magnetic field inhomogeneity near the superparamagnetic nanoparticles to detect magnetically labeled cells is to form images based on quantitative measurement of the magnetic susceptibility, or quantitative susceptibility mapping (QSM; Wang and Liu, 2015). As normal tissue has much lower magnetic susceptibility than iron oxide nanoparticles, nanoparticle-laden cells can be readily distinguished using this approach. An added advantage is the ability to discriminate the iron oxide MRI signal from other structures that cause dark contrast such as air and blood vessels (Haacke et al., 2015). Other techniques negate the magnetic susceptibility effect and generate images principally characterized by T1 effects of the iron oxide nanoparticles. These pulse sequences, such as ultrashort echo time (UTE; Hong et al., 2017) and SWeep Imaging with Fourier Transformation (SWIFT; Magnitsky et al., 2017, 2018), use very short echo times to prevent dephasing of the excited protons, capturing the signal from the water near the nanoparticles, and instead use T1 weighting to create hypointensity at the spot of the labeled cells. The exciting nature of the pulse sequences described in this paragraph is the quantitative nature of these sequences, enabling the *in vivo* measurement of iron concentrations (Ring et al., 2018). These types of pulse sequences and data analyses might be most useful for determining the relative number of cells in a large cell transplant, for example.

The above-mentioned pulse sequences enable measurement of iron concentrations, but the conversion of iron concentration to cell number is not straightforward as individual cells can have different amounts of iron. The use of very high resolution *in vivo* MRI is one approach to solving the challenge of cell enumeration. MRI detection of single cells has been demonstrated by employing GRE based techniques (Shapiro et al., 2006b). Mills et al. (2008, 2011) used phase map cross correlation to discriminate individual magnetically labeled immune cells in rat heart and brain. Mori et al. (2014) used high resolution GRE images to detect and enumerate magnetically labeled microglia in the brain, making use of signal thresholding to identify cells. Afridi et al. (2017), acquired GRE images of rat brain following injection of magnetically labeled mesenchymal stem cells, where individual cells were visible as dark spots. Machine learning was used to non-invasively quantify the number of cells that were delivered to the brain. Whereas not all cell transplant or stem cell migration paradigms are amenable to this type of analysis, measurement of cell number is clearly an open area of research in the field of MRI-based cell tracking of neural cells.

## MRI of Endogenous NPCs

Magnetic resonance imaging of endogenous NPCs is accomplished by magnetically labeling proliferating NPCs *in situ* and using MRI to serially monitor migration away from the SVZ; in healthy animals, cells travel along the RMS to the OB, however, disease stimulates these cells to migrate away from the RMS and toward lesions in the brain. In this section, we will describe how the work on this elegant technique for tracking migrating cells has focused on developing and improving the labeling methods and determining which particles offer the best labeling and imaging, with the large MPIO particles offering the best detection of migrating NPC. We will discuss the application of labeling of endogenous NPC to the study of disease models such as multiple sclerosis and neonatal hypoxia-ischemia. Another option for tracking endogenous NPCs is to transfect them with one of the recently described MRI reporter genes; the genes used in this emerging technique and the opportunities they enable will be discussed below.

### *In situ* Labeling Methods

**FIGURE 2:**
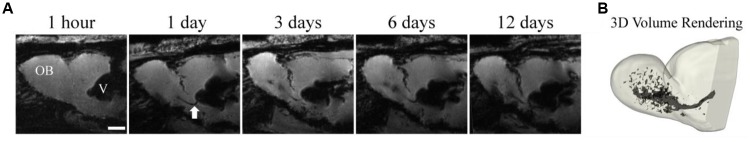
Temporal resolution during early migratory events. MPIOs (50 μL) were injected into the anterior horn of the lateral ventricle proximal to the SVZ in healthy adult rats. 3D gradient echo images of a rat brain were serially acquired at 1 h, 1 days, 3 days, 6 days, and 12 days **(A)** post injection. The RMS is visible on the day 1 images as a dark line extending from the ventricle (white arrow). Migration across the entire OB is visible by day 12, the 3D volume rendering of the OB **(B)** shows the extent of cells within the structure (Adapted from Shuboni-Mulligan et al., 2018).

*In situ* labeling of NPCs with iron oxides first involved injecting very small ∼20 nm particles into the carotid artery, then disrupting the blood-brain barrier (BBB) with mannitol to allow the particles to enter the brain and label NPC (Neuwelt et al., 1994). Shapiro et al. (2006a) developed the technique of injecting MPIOs in a volume of 10–50 μL of stock particles (1.63 μm diameter particles, ∼3 μg Fe/μL) into the anterior horn of the lateral ventricle to label NPCs and track them over time in their migration through the RMS to the OB. This foundational work has been the basis for endogenous NPC labeling tracking studies. Figure 2 shows an example of migrating cells in the RMS and OB after injection of MPIO particles into the SVZ. The literature since has used several different doses and coordinates (Table 1). Granot et al. (2011) attempted to further optimize *in situ* labeling and found that a more rostral injection site and a smaller dose of 20 μL of MPIOs produced the best labeling of NPCs in the RMS, however, in a later publication the same authors used different, more rostral coordinates (Granot and Shapiro, 2014).

**Table 1:** *In situ* NPC labeling technique.

Citation	Species/strain	Age	Injection Volume	Coordinates		
				AP	LM	DV
Shapiro et al., 2006a	Sprague-Dawley	6 weeks	5–50 μL	-2.0 mm	2.0 mm	-3.0 mm
Sumner et al., 2007	Sprague-Dawley	6 weeks	50 μL	-2.0 mm	2.0 mm	-3.0mm
Sumner et al., 2009	Rat/Sprague-Dawley	6 weeks	50 μL	-2.0 mm	2.0 mm	-3.0 mm
Panizzo et al., 2009	Rat/Sprague-Dawley	Not given	20 μL	-0.08 mm	0.14 mm	-0.38 mm
Vreys et al., 2010	Mouse/C57BL/6J	Not given	10 or 1.5 μL	0.3 or 1.7 mm	1 or 0.7 mm	-2.3 or 4.1 mm
Nieman et al., 2010	Mouse/ICR	6–10 weeks	50 nl	-1.2 mm	0.8 mm	-2.5 mm
Granot et al., 2011	Rat/Sprague-Dawley	6 weeks	20–30 μL	-1.5 or -2 mm	1.5 or 2 mm	-3.0 mm
Granot et al., 2014	Rat/Sprague-Dawley	Not given	20 μL	-1.5 mm	1.5 mm	-3.0 mm
Granot and Shapiro, 2014	Rat/Sprague-Dawley	Not given	20 μL	-0.8 mm	1.2 mm	-4.0 mm
Pothayee et al., 2017	Rat/Sprague-Dawley	6 weeks	20 μL	1.9–2.0 mm	1.9–2.0 mm	-4.0 mm
Shuboni-Mulligan et al., 2018	Rat/Fischer 344	6 weeks–2 years	50 μL	0.3 mm	1 mm	-3.5 mm

Smaller particles were less effective for monitoring migration in the RMS and OB. The clinically approved SPIO called Endorem has a particle size of 120–180 nm, and these particles were evaluated for NPC labeling after injection into the ventricles of healthy rats either with, or without, co-injection of protamine sulfate at a dose of ∼7 μg Fe/μL in a volume of 2.5 μL (Panizzo et al., 2009). The protamine sulfate-Endorem complexes labeled the NPC *in situ* while the uncomplexed Endorem did not, and in fact, was shown to spread throughout the ventricles after injection. Migrating cells labeled with these smaller Endorem particles after co-injection with protamine sulfate were visible in the RMS, but not strongly, when imaged *in situ* at 2.35T, and were not visible in the OB. *Ex vivo* imaging at 9.4T was needed to visualize NPC in the OB after labeling with Endorem particles (Panizzo et al., 2009).

Transfection agents have also been used to assist *in situ* cell labeling with MPIOs. In healthy mice, co-injection of 1.5 μL of a mixture of MPIOs and PLL (∼0.67 μg Fe/μL) provided better labeling than an injection of MPIOs and saline in the same volume. Use of the transfection reagent led to good labeling that enabled visualization of cells in the RMS and there seemed to be minimal effects of the labeling on NPC proliferation and no significant inflammation. A higher dose (10 μL, ∼3 μg Fe/μL) of MPIOs did reduce NPC proliferation (Vreys et al., 2010; Guglielmetti et al., 2014).

### Specificity of *in situ* Cell Labeling

When MPIOs or SPIOs are injected into the lateral ventricle, uptake of the label is not restricted only to NPCs – scavenger cells and other non-migrating cells can also internalize the particles along the ventricles and choroid plexus. Migrating cells in the RMS can also be ‘pruned’ (Brunjes and Armstrong, 1996; Winner et al., 2002) and the label may be taken up by microglia during this process. This could lead to signals in the MRI image that are not associated with NPCs. The extent and significance of these nonspecific signals has been addressed in a number of studies. Sumner et al. (2009) used flow cytometry to determine the relative proportion of labeled cell types in excised and digested RMS and OB. At 2 weeks post-injection (50 μL, 3 μg Fe/μL) they observed that ∼40% of the labeled cells were astrocytes, ∼30% oligodendrocytes, ∼30% neurons, with ∼5% microglia in these excised samples of the RMS and SVZ. In the OB, the fraction of astrocytes remained the same, but the proportion of oligodendrocytes increased slightly and neurons decreased to 10% of labeled cells, with a greater percent of microglia (over 10%; Sumner et al., 2009). In contrast, another study in mice using histological staining found that 2 days after labeling with 1.63 μm particles (50 nL, 3 μg Fe/μL), the label was predominantly confined to migrating NPCs (∼60%) and astrocytes (∼25%), but after 21 days, up to 35% of labeled cells were microglia instead of migrating cells, with NPCs remaining at ∼50% and astrocytes ∼25%; there was no difference between the labeled populations in the RMS and the OB (Nieman et al., 2010). This suggests that as the experiment progresses, the label is less likely to be inside migrating cells derived from NPCs. One suggestion is that labeled microglia can be discriminated from labeled NPCs because the microglia will be stationary, while the NPCs will be moving (Shapiro et al., 2006a), though this experiment would be time consuming, requiring multiple scans.

Migration of free particles could also be a problem and is not easily differentiated from labeled cells by MRI. Sumner et al. (2009) found that MPIOs did not migrate spontaneously along the RMS but needed to be carried by a migrating cell. In another experiment, cortical stroke was induced in rats by the injection of ET1, followed by injection of 20 μL of MPIO (0.86 μm, 2 μg Fe/μL). Signal voids were seen at the site of the stroke; however, they were not caused by labeled NPCs, but were due to migration of free particles that were carried by the CSF through the corpus callosum toward the lesion. In this model, the ET1 also acts to disrupt the blood–brain barrier and allow the influx of particles into the brain, which is not a concern in healthy animals, but this does indicate that the location of the lesion must be chosen carefully to avoid the migration of free particles (Granot and Shapiro, 2014). In another study, when magnetoliposomes were injected into the RMS of mice (1.5 μL at 0.67 μg Fe/μL), free magnetoliposomes were seen in white matter tracts and in the RMS but were rarely seen encapsulated in migrating cells (Vreys et al., 2011). They also observed that there was less free motion of cationic magnetoliposomes than anionic ones, so the authors concluded that it may be possible to use larger cationic magnetoliposomes to label NPCs without the risk of migration of free particles (Vreys et al., 2011).

**FIGURE 3:**
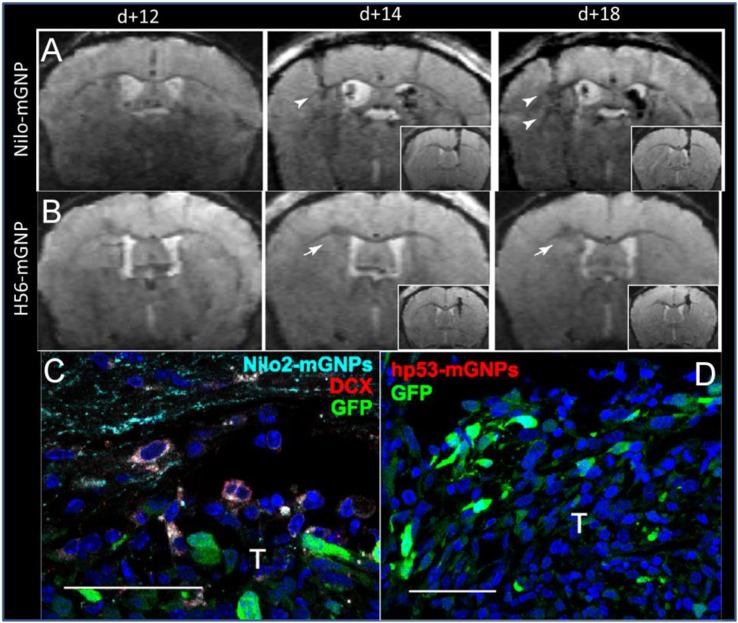
Targeted magnetic glyconanoparticles (mGNP) specifically labeled NPC and the NPC migrated to astrocytomas. mGNP were fabricated with **(A)** antibodies that target NPC (Nilo) or **(B)** control antibodies (H56) and injected contralaterally to CT-2A astrocytoma on day 13 of tumor growth. Arrowheads indicate hypointense signal at the tumors as early as 1 day after NPC injection, while arrows indicate absence of hypointense signals. No control particles were detected at the tumor. **(C)** NPC (red) were labeled with the mGNP (light blue) and migrated to the tumor (green). **(D)** No control nanoparticle labeled cells were detected near the tumor. T, tumor, scale bar is 50 μm (Elvira et al., 2012). Reproduced via Plos One Creative Commons Attribution (CC BY) license.

Elvira et al. (2012) made targeted magnetic glyconanoparticles (4 nm diameter) conjugated to Nilo2, an antibody directed against NPCs. These particles were injected into the contralateral ventricle of mice with astrocytoma (1 μL of particles at 0.1 μg Fe/μL). One day after injection, contrast was seen in the tumors as the NPCs at the site of the particle injection migrated to the tumor; indeed, most of the neuroblasts at the tumor were labeled. When a non-targeted particle was injected, there was no accumulation of contrast at the tumor, although NPCs were present (Figure 3). Another study used SPIOs conjugated to CD15 to label *in situ* NPCs (Zhong et al., 2015), increasing the labeling efficiency while decreasing distortions caused by direct MPIO injection. The authors go on to demonstrate the effectiveness of the technique using a stroke model (Zhang et al., 2016). These studies show the potential of using small particles for MR tracking of NPCs in the situation where large numbers of labeled cells are expected to be localized in the brain. It is anticipated that the use of smaller magnetic particles would not have allowed for single-cell detection if the labeled cells were sparsely distributed.

### Image Analysis Methods

The contrast due to iron labeled cells is visually apparent for qualitative analysis, although there can be some ambiguity with respect to other hypointense structures in the brain; these depend on the acquisition parameters. Some more complex image analysis methods have also been used to quantify the signal or to discern more subtle or time-dependent effects. Sumner et al. (2009) plotted signal over a line segment to show that the signal in the RMS dropped 60% from the background when there were migrating labeled cells present, while similar studies in phantoms from the same group found that a single cell labeled with MPIOs would cause a 30% signal drop (Shapiro et al., 2005). Subsequently, Granot et al. (2011) registered images of rat brains pre- and post-MPIO injection and looked for regions with a signal decrease of >30% to quantify the migration of cells in the OB. Using this method, they were able to quantify the volume of the OB that was occupied by signal from migrating cells. This signal appeared 2–3 days after MPIO injection, increased linearly until day 7, and then plateaued (Granot et al., 2011). Nieman et al. (2010) used image registration and intensity-based analysis to identify labeled cells and determine their speed of migration. They estimated that labeled NPCs move at 100–120 μm/h in the RMS and 50 μm/h in the OB (Nieman et al., 2010; Pothayee et al., 2017; Shuboni-Mulligan et al., 2018), slightly slower than the speeds of 150–700 μm/h that were measured in mice with tumors (Elvira et al., 2012). These migration rates are not far from those determined in studies of neurogenesis that employ immunohistochemical methods of detection, which determined a migration rate of 70–80 μm/h in naïve rodents (Nam et al., 2007). Recently, the technique demonstrated that both environmental stimuli, olfactory activity (Pothayee et al., 2017) and aging (Shuboni-Mulligan et al., 2018) impacted migration rates *in vivo*. Understanding the dramatic decrease in NPC migration as animals aged is critical, as many diseases that recruit NPC are more prevalent in older individuals (Wang et al., 2013).

### Applications of Endogenous NPCs MRI

Magnetic resonance imaging of endogenous NPCs has also been applied in brain diseases and disorders where there is a need for regeneration of neurons. In neonatal rats with hypoxia-ischemia caused by a ligation for 5 min of the left common carotid artery and then placement into hypoxic chamber (8% O_2_ and 92% N_2_) for 2 h, MPIOs (10 μL of 0.86 μm particles, 2 μg Fe/μL) were injected into the ventricles to label NPCs. As expected, labeled NPCs migrated through the RMS in healthy rats, but a small portion also migrated toward ischemic regions in the hypoxic-ischemic rats (Yang et al., 2009). The therapeutic effect of the migrating cells was examined using endogenous NPC from the SVZ in a study labeling cells with intraventricular injection of anti-CD15 SPIOs. Proliferation of NPCs in the SVZ was blocked using Ara-A infusion, which ceased migration of cells and prevented the decrease in infarct size over time (Zhang et al., 2016). In another experiment, NPC were labeled with MPIOs (1.63 μm particles, 1.5 μL, 0.67 μg Fe/μL) plus PLL to see if NPC from the SVZ were the source of remyelination following cuprizone treatment in a mouse model of MS. There was no migration of cells from the SVZ to the splenium, so the origin of the new cells was not determined (Guglielmetti et al., 2014).

### Reporter Genes in NPCs

Magnetic resonance imaging reporter genes can be used to induce cells to produce proteins that create MRI contrast; one example of such a protein is the iron storage protein, ferritin. Cell tracking with ferritin-overexpressing cells would allow for specific imaging of NPCs without confounding images from label uptake by microglia or free particles, and the label would not be lost through cell division. These are two properties that comprise the strengths of reporter genes, however, the limitation is the requirement of genetically engineering the cells. Ferritin consists of a light chain (L) and a heavy chain (H) that form a shell that can be filled with excess intracellular iron. A ferritin transgene (H and L chains) in an adenoviral vector was injected into the striatum of mice and a clear signal loss was seen at the injection site as soon as 5 days post-transplant from transfected glia and neurons (Genove et al., 2005). The specificity and sensitivity of detecting the ferritin gene following transfection via lentiviral and adenoviral vectors has been assessed (Vande Velde et al., 2011). In this study, the control lentivirus, with the gene encoding green fluorescent protein (GFP), caused a hypointense signal in the brain that was equivalent to the contrast induced when the lentivirus containing the gene for ferritin was used; this was due to an inflammatory response (Vande Velde et al., 2011). This inflammatory response was not seen when an adenovirus construct was used in the same study. A further study injected the lentiviral vector with the ferritin gene into the SVZ or striatum of mice. Despite extensive correction of the RF field bias, there was only minimal signal from migrating NPCs, and it required T_2_^∗^ mapping (Vande Velde et al., 2012). Lentiviral transfection with ferritin of exogenous NPCs could be visualized with MRI and fluorescence imaging when reintroduced intracranially into rats with acute ischemic stroke but also not of the migration of single cells (Zhang et al., 2017).

A different adenovirus encoding the L^∗^H chains of ferritin has also been injected into mice and in this study, some astrocytes and neurons were transfected (Iordanova et al., 2010; Iordanova and Ahrens, 2012). Some of the transfected cells in the SVZ were proliferative as indicated by Ki67 staining, but transfection was not specific to proliferative cells. In the OB, transfected cells were visible by histology, but not by MRI (Iordanova and Ahrens, 2012). Sensitivity was quantified as 10^4^ cells per voxel. A follow-up study used the neurotrophic HSV virus, which was thought to target neurons selectively over glia. In this study, the amount of signal correlated with the amount of virus injected, and this study supports the use of the ferritin as a means to quantify gene delivery in nucleic acid based therapies, rather than a method for tracking the migration of individual transfected neurons (Iordanova et al., 2013). Further modifications to the genetic construct may offer more functionality, and toward this end, inducible ferritin constructs have been used in culture to monitor the differentiation of mesenchymal stem cells into neural cells (Song et al., 2015).

## Mri of Exogenously Administered NPCs

An alternative method to visualize iron labeled NPCs is to label the cells exogenously and reintroduce them back into an animal. For several disease models, exogenously introduced NPCs have been shown to have a capability to home to areas of disease or injury. There are two distinct applications for monitoring the exogenous administration of NPCs: (1) the monitoring of integrated cells in regenerative medicine and (2) the identification of cell migration to disease area for treatment with modified cells. These applications will be examined here in the context of stroke and glioma.

### *In vitro* NPC Labeling Methods

There are various sources of NPC that can be used for this purpose (Table 2): they can be isolated from the SVZ of adult rodents (e.g., Zhang et al., 2003; Jiang et al., 2005), immortalized cell lines isolated from embryonic rodent brain (e.g., Hoehn et al., 2002; Guzman et al., 2008; Obenaus et al., 2011), and finally human NPC can be isolated from human fetal tissue (e.g., Daadi et al., 2013; Ashwal et al., 2014; Song et al., 2015; Barish et al., 2017). Feridex has been the most common particle used for labeling NPCs in culture (Nucci et al., 2015). Typical iron concentrations for magnetic cell labeling range from 11.2 μg Fe/mL (Ashwal et al., 2014) to 112.3 μg Fe/mL (Song et al., 2009). The simplest method of labeling is incubating cells with particles for 3 days (Song et al., 2009) or for 1 day following trypsinization (Ashwal et al., 2014). Labeling cells with iron oxide particles using these methods had no significant effect on cell viability or on ability to differentiate and migrate (Ben-Hur et al., 2007), especially at low (but still MR-visible) doses. However, some studies with mesenchymal stem cells (Bulte et al., 2004) have found differentiation along some lineages can be impaired; other studies have seen no effect (Arbab et al., 2005b; Kassis et al., 2010). Each cell type that is labeled in this way should be evaluated for effects on cell function prior to conducting studies *in vivo*.

**Table 2:** Exogenous neural progenitor cells in stroke.

Citation	Rat breed	Cell type	SPIO	Cell origin	Injection type
**(a) Mouse neural stem cells (mNSC)**					
Hoehn et al., 2002	Wistar	HT22 mNSC	Ferumoxtran-10, Sinerem^®^	Immortalized primary mouse hippocampal neural cells derived from parent HT4 cells	Intracranial – Corpus Callosum/Cortex border
Modo et al., 2004	Sprague-Dawley	MHP36 mNSC	Gadolinium-Rhodamine Dextran (GRID)	Cell line isolated from hippocampal proliferative zone of fetal mice (H-2K^b^-tsA58)	Intracranial -Striatum
Modo et al., 2009	Sprague-Dawley	MHP36 mNSC	Gadolinium-Rhodamine Dextran (GRID)	Cell line isolated from hippocampal proliferative zone of fetal mice (H-2K^b^-tsA58)	Intracranial -Striatum
Guzman et al., 2008	Sprague-Dawley	C17.2 mNSC	Ferumoxides, Feridex^®^IV	Immortalized cell line established from neonatal mouse (CD1 × C57BL/6) cerebellum	Intracranial – Cortex
Obenaus et al., 2011	Sprague -Dawley	C17.2 mNSC	Ferumoxides, Feridex^®^IV	Immortalized cell line established from neonatal mouse (CD1 × C57BL/6) cerebellum	Intracranial – Striatum or Intraventricular – Lateral Ventricle
**(b) Rat neural stem cells (rNSC)**					
Zhang et al., 2003	Wistar	rNSC	MPIO, Bangs	Isolated rNSC from young adult rat brain (SVZ)	Intracistern -Cisterna Magna
Jiang et al., 2005	Wistar	rNSC	MPIO, Bangs	Isolated rNSC from the lateral wall of the lateral ventricle of adult Wistar rat	Intracistern -Cisterna Magna
Li et al., 2006	Wistar	rNSC	MPIO, Bangs	Isolated rNSC from the adult rat brain near the lateral ventricle	Intracistern -Cisterna Magna
Li et al., 2010	Wistar	rNSC	Ferumoxides, Feridex^®^IV	Isolated rNSC from young adult rat brain (SVZ)	
Wen et al., 2014	Sprague-Dawley	rNSC	Polymersomes PEI-PDDLA	Isolated rNSC from the new born rat brain near the lateral ventricle	Intracranial -Striatum
**(c) Human neural stem Cells (hNSC)**					
Daadi et al., 2009	Sprague-Dawley	hNSC	Ferumoxides, Feridex^®^IV	H7 Human embryonic stem cell (WiCell) derived with supplemented media	Intracranial -Striatum
Daadi et al., 2013	Sprague-Dawley	hNSC	Ferumoxides, Feridex^®^IV	H7 Human embryonic stem cell (WiCell) derived with supplemented media	Intracranial -Striatum
Song et al., 2009	Sprague-Dawley	HB1.F3 hNSC	Ferumoxides, Feridex^®^IV	Immortalized hNSC established from human fetal brain (ventricular zone)	Intracranial – Striatum or Intravascular – IV
Ashwal et al., 2014	Sprague-Dawley	HFB 2050 hNSC	Ferumoxides, Feridex^®^IV	Cell line isolated from the ventricular zone of a human fetal cadaver	Intraventricular – Lateral Ventricle

### Applications of Exogenous NPCs in Regenerative Medicine

The aim of regenerative medicine is to repair damaged tissues or organs within an organism through replacement or restoration (Mason and Dunnill, 2008). Stem cells, such as NPCs in the brain, play an important role in regeneration and have been evaluated in several disease and injury models. These cell replacement techniques have been paired with MRI in the investigation of recovery from spinal cord injury (Lepore et al., 2006), reducing autoimmune damage in multiple sclerosis (Ben-Hur et al., 2007; Reekmans et al., 2011), and to restore function in stroke models (Hoehn et al., 2002; Zhang et al., 2003).

The disease that has been the most extensively researched using exogenously labeled NPCs and MRI is stroke. In the United States, stroke is the fourth leading cause of death and occurs in one out of every 19 fatalities (Kochanek et al., 2011). Over the past 10 years, stroke-attributed deaths have decreased (Go et al., 2014) thus increasing the demand for regenerative medicine treatment of survivors, who suffer from the adverse effects of stroke. Promising data has been collected in both animal models and clinical trials using exogenous NPCs to reverse or repair damage induced by stroke. Incorporation of MR imaging into the evaluation of NPCs as cell-based therapies is a way for researchers to gain an understanding of the relevant cellular processes, and to guide the development of this approach into a viable strategy for effective regenerative medicine.

**FIGURE 4:**
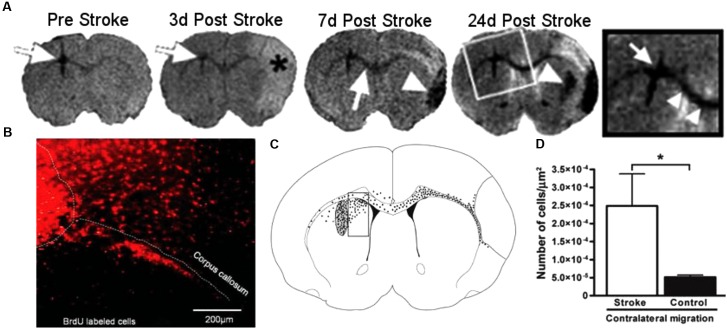
Magnetic resonance imaging (MRI) of NPCs migrating to stroke (Guzman et al., 2008, reproduced with permission). The top panel **(A)** shows a representative MRI of animals injected with NPCs before and over 24 days after the induction of stroke. White arrows show the location of labeled cells, moving from the site of injection to the area of stroke, denoted with an asterisk (^∗^). The bottom panel **(B–D)** shows the histology of animals 24 days after stroke. Cells labeled with BrdU (red, **B**) were observed migrating away from the site of injection and into the corpus callosum. To quantify the migration, the area adjacent to the injection site was analyzed by counting cells in a counting box **(C)**. The graph shows significantly greater migration away from the injection site in animals that received a stroke than the sham controls. Reproduced with permission.

**FIGURE 5:**
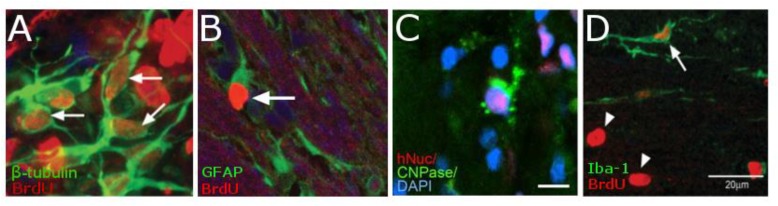
Histology demonstrating the cell types of NPCs after differentiation *in vivo*. NPCs are double-labeled with BrdU (red) and either β-tubulin (green, **A**) showing that cells are differentiation into neurons. Cells that have differentiated into astrocytes are double-labeled with BrdU (red) and GFAP (Green, **B**). While those cells that have differentiated into oligodendrocytes are triple-labeled for hNSC (red), CNPase (green), and the nucleus with DAPI (blue **C**). However, there is very little macrophages-positive staining (OX6, Green) that is co-localized for BrdU (red, **D**). [Data from Guzman et al., 2008, reproduced with permission); Daadi et al., 2008, Reproduced via Plos One Creative Commons Attribution (CC BY) license].

In rodent models of ischemic stroke, where the middle cerebral artery (MCAO) is occluded, administration of NPCs has led to marked improvements in both behavioral and physiological markers of animals. The use of iron oxide particles to label cells has allowed researchers to use MRI to visualize the movement and incorporation of cells into the site of stroke (Hoehn et al., 2002; Zhang et al., 2003) and has demonstrated that the migration is directly triggered by the onset of disease (Figure 4; Guzman et al., 2008). Additionally, many studies have established that of exogenous NPCs labeled with magnetic particles retain normal function with the same positive effect on restoration of function as control cells without particles. In these studies, magnetically labeled NPCs have been shown to reduce the size of the lesion (Obenaus et al., 2011; Daadi et al., 2013), increase angiogenesis (Jiang et al., 2005; Li et al., 2006) and improve glucose utilization (Daadi et al., 2013) in damaged areas of the brain, and recover sensorimotor functions (Zhang et al., 2003; Daadi et al., 2009). In a large animal, porcine, model of ischemia, NPCs injected into the region of stroke decrease the immune response and reduced changes in cerebral blood perfusion and brain metabolism (Baker et al., 2017). Additionally, rodent studies have demonstrated that labeled cells that migrated into the site of the infarct have functionally incorporated into the tissue (Figure 5) and differentiated into neurons (β-tubulin and NeuN), astrocytes (GFAP), and oligodendrocytes (CNPase) (Song et al., 2009; Daadi et al., 2013). These cells did not, however, counterstain with antibodies that were specific for microglia and macrophages (OX6; Song et al., 2009). The cellular and molecular mechanisms that lead to functional improvement have not yet been established, although several hypotheses have been proposed (reviewed in Boese et al., 2018). The effects could be mediated by replacement of lost circuitry, neuroprotective function, improved vascular recovery, paracrine stimulation of surviving neurons and other cells, recruitment of endogenous progenitors, or reduced inflammation.

### Factors Affecting MRI of Exogenous NPCs

Implementation of MR imaging of cellular fates in stroke research has the potential for making NPC transplantation a realistic therapy for patients suffering from this disease. Through imaging we can refine our understanding of the optimal route and timing of injection, the effective dose of cells for a given lesion, rapid determination of lesion size, and the relevant cell types for reversing the damage and restoring function. In stroke, four routes of administration of cells have been used: intracerebral, intraventricular, intracisternal, and intravascular. The most common route used in animal studies is intra-cerebral injections into the striatum (Table 2) and this is also being evaluated in clinical trials. Investigation in the field of regenerative medicine originally focused on this approach because researchers wanted to limit the distance cells would have to migrate and eliminate the complications of requiring cells to penetrate the blood brain barrier (Willing and Shahaduzzaman, 2013). However, intra-cerebral injection is an invasive procedure that involves complex stereotactic surgery in patients already weakened by stroke; therefore, other routes of administration would be preferable. Intra-ventricular and intracisternal injections are less invasive and may minimize tissue damage by targeting the brain ventricles or subarachnoid spaces, respectively. Both methods have been demonstrated to allow cell migration to the site of stroke (Li et al., 2006; Obenaus et al., 2011; Ashwal et al., 2014); however, only one study compared these methods directly to intracerebral injection. In this study (Obenaus et al., 2011), more cellular migration was observed with intra-ventricular injections compared to intracerebral injections into the striatum, however, no statistics or compelling images were provided to bolster these claims.

The least invasive routes of NPCs administration are peripheral injections, either venous (IV; Song et al., 2009) or arterial (IA; Li et al., 2010). When comparing these two vascular routes of administration to intracisternal injections, Li et al. (2010) found that IA injections had the fastest and greatest dispersion of cells within the stroke area while IV injection had the slowest and least number of cells, however, animals that received IA injections also had a higher mortality. This study, while providing interesting insights into the migration of NPCs after these injection routes, did not address the full range of physiological effects, the overall localization of cells, or the cause of mortality. More in depth investigation will be required prior to drawing any conclusions and developing an effective treatment plan for patients is identified to minimize complications while providing optimal care.

The relationship between cell dose and recovery is more straight-forward. When heNSCs labeled with Feridex were injected into the striatum of rats with stroke, the number of cells administered was inversely proportional to the size of the stroke after treatment (Daadi et al., 2009, 2013). Thus, studies comparing injection routes should control for number of cells to avoid confounding results. Additionally, the relationship between the number of transplanted cells and stroke recovery is even more complicated. The more severe and larger the initial stroke volume, the less of an impact treatment with NPCs has on the outcome of adult (Daadi et al., 2013) and infant (Ashwal et al., 2014) animals with stroke. Others have determined that, paradoxically, a lower dose of NPC is more effective at achieving homing toward a tumor (Barish et al., 2017).

There are still many questions that have yet to be addressed with these models; including the best cell type and the best methodology for labeling cells, since a wide variety of each have been used, as well as, the interaction of the transplanted cells with those of the tissue. Rodent studies have used cells isolated from the SVZ of adult rats, immortalized NPC lines from rodent embryos, or those isolated from human fetuses. In humans, there are two abstracts that have used NPCs derived from human fetal tissue and demonstrate improved symptoms in patients (Kalladka et al., 2013; Qiao et al., 2014). The authors of one abstract are currently running two clinical trials (NCT01151124 and NCT02117635, ClinicalTrial.gov) using the cell line CTX0E03 DP, an immortalized human stem cell line derived from third trimester human fetal cortex (Pollock et al., 2006). These different types of cells have never been compared in terms of efficiency; therefore, generalizations between studies should be analyzed in this light. The phase I escalation trial showed no adverse effects of the cell injection and modest neurological improvements in some patients (Kalladka et al., 2016), the phase II trial has completed recruitment, but no results have been published.

### Applications of Exogenous NPCs in Glioma Therapies

Another application of magnetically labeled exogenous NPCs is their use in the detection or targeted treatment of glioma. Gliomas arise within the nervous system and are classified by histopathology and immunohistochemistry (Tsankova and Canoll, 2014). GBM is a particularly aggressive primary brain tumor with a grim prognosis for patients; the average lifespan is less than 12 months (Towner et al., 2011). Because the brain is an immune-privileged area within the body and is protected by the blood–brain barrier, detection and treatment of these tumors presents a particular challenge. Research into the use of NPCs in the treatment of glioma has made progress in harnessing the unique characteristics of NPCs in migrating toward glioma cells and the power of cell tracking via MRI.

**FIGURE 6:**
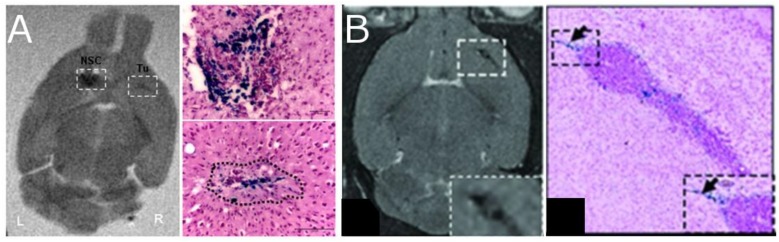
Neural progenitor cells (NPCs) migrating to glioma after injection of SPIO labeled cells. The left two panels **(A)** show coronal *ex vivo* MRI images (T_2_-weighted) and Prussian Blue staining of tumor in animals with tumors 4 days after contralateral intracranial NPCs injection [Thu et al., 2009, reproduced via Plos One Creative Commons Attribution (CC BY) license]. Cells migrate away from the injection site and into the tumor. The right two panels **(B)** show *in vivo* MRI images (T_2_-weighted) and the Prussian blue histology of animals with IV injections of NPC (Gutova et al., 2013, reproduced with permission).

Early studies used histological methods to demonstrate that (1) NPCs actively migrate into the tumor itself (Aboody et al., 2000) and (2) the mere presence of NPCs could act to impede the growth and proliferation of tumors (Glass et al., 2005). Both results were later replicated using MRI-based cell tracking. Brekke et al. (2007) used gadolinium-based particles to determine that NPCs injected contralateral to the tumor site migrated across the corpus callosum to the site of the tumor (Figure 6) and that tumor doubling time was significantly decreased, probably due to reduced edema formation (Brekke et al., 2007). Others have explored different routes of administration and found migration to the tumor following IV (Gutova et al., 2013), intracisternal (Zhang et al., 2004) and intracerebral (Thu et al., 2009; Chaumeil et al., 2012) injections of NPCs, demonstrating that cells also penetrate the tumor with these routes. The effects of NPC presence on tumor growth, however, were trivial and did not greatly alter the course of the disease.

The next step in the progression of the NPCs/glioma research focused on modifying NPCs to deliver agents to the site of the tumor and have a greater impact on tumor growth. Cells can be altered to (1) deliver specific cytokines, (2) impact angiogenesis, or (3) to function as “suicide cells” that deliver prodrugs to the effective area (Kwiatkowska et al., 2013). MRI labeling of NPCs with MPIOs has been used in prodrug delivery studies to optimize the timing of the activation of the suicide cell. Researchers have established cell lines that can produce an enzyme that can convert a prodrug into a chemotherapeutic agent for the purpose of directed delivery (Aboody et al., 2013). The effectiveness of this method has been verified in combination with iron labeling to demonstrate the advantages of using the combination, and patients were recruited into a phase I clinical trial (NCT01172964, ClinicalTrial.gov). This trial has completed their recruitment, and while no results have been published, two additional trials have been launched (NCT02015819 and NCT02192359) to test the addition of the combination of Leucovorin or to modify cells to sensitize them to the drug Irinotecan (Mutukula and Elkabetz, 2017).

Another novel approach in the use of MRI and magnetically labeled NPCs in the treatment of GBM is their possible use in the detection of tumor recurrence. Recurrence of tumors in GBM patients is a universal trait of the disease and is believed to be a major contributor to the poor survival of patients (Park et al., 2010). New treatments designed to reduce tumor size have been shown to increase the migration of tumor cells and the formation of recurrent masses further from the original site (Ogura et al., 2013). One suggestion is to use MPIO-labeled stem cells as a diagnostic tool for identifying small masses of tumor (<1 mm^2^) that are not detectible with current MRI methods (Bennewitz et al., 2012). While the authors suggested the use of mesenchymal stem cells, NPCs might provide a more attractive alternative. Indeed, there is already some anecdotal data showing the ability of NPCs to detect individual glioma cells (Zhang et al., 2004), however, this is based on immunohistochemistry demonstrating a single NPC near a glioma cell within the corpus callosum. This hypothesis merits further investigation but it is imperative to examine the signals that recruit cells to the tumor and the ability of NPCs to home to early tumors and micro-metastases in a more prospectively designed experiment.

Additionally, others suggested the use of NPCs as a method to infect remaining tumor cells with a conditionally replicative adenovirus after surgical resection of the mass. In a preliminary study, Morshed et al. (2015) imaged NPCs carrying a cytotoxic adenovirus infiltrating the margins of the tumor. This study is an initial step but shows a promising new avenue for the combination of MRI and NPCs in the treatment of GBM.

### Obstacles for Tracking Exogenously Labeled NPCs

Beyond the lack of consistency with regards to techniques for cell administration in the preclinical and clinical studies mentioned above, the tracking of exogenously labeled NPCs is limited by factors related to health of the injected cells and the persistence of the loaded particles in cells after transfer. Selection of cells and particles within an experiment is, therefore, an important component of the quality control in tracking studies.

The health of cells being injected into patients is important for the transfer of therapeutic effect as well as for both the short and long-term ability to monitoring cells *in vivo* over time. Immediately following injection of cells, there is a possibility of the grafted cells being rejected, and this has been examined in a handful of models. Mesenchymal stem cells (MSC) can be obtained from patient and the use of such autologous grafts should reduce the likelihood of rejection (Akgun et al., 2015). MSC can be harvested in the operating room, cultured and labeled, then reintroduced into the targeted area of the patient (Callera and de Melo, 2007; Kassis et al., 2010). The importance of using autologous MSCs has not been definitively established (Kode et al., 2009). For ischemic cardiomyopathy, allogeneic, and autologous MSC produced similar positive therapeutic results, and neither triggered an immune response (Hare et al., 2012). In contrast, NPCs cannot easily be harvested from patients; therefore, these cells are collected from donors, i.e., fetal cadavers. The sex of the NPC donor was found not to be relevant to the success of the graft in a rat study (Ashwal et al., 2014), however, other parameters have not been fully examined. Taken together these studies suggest that the risk of rejection is low. However, a recent article, Bago et al. (2017) demonstrated the possibility of using donor fibroblasts to *trans*-differentiate into NSCs and allowing for autologous cells to be cultured into cytotoxic agents against glioblastoma.

A major criticism of using iron oxide nanoparticles for cell tracking is that the signal is retained even after the labeled cells are no longer viable. MRI does, however, have the potential to differentiate between live and dead cells. In immunosuppressed rats, there was a clear difference in the MRI appearance and image intensity and graft size over time between live and dead NPCs (Guzman et al., 2007). When live cells were injected, the size of the hypointensity was constant with time, while transfer of dead cells led to a decrease (Guzman et al., 2007). In a different study of immunocompetent rats, live cells were observed to migrate leading to an increase in the size of the hypointense region, and since dead cells don’t migrate the size of the hypointense region remained constant (Flexman et al., 2011). In a model where animals rejected grafted cells, hypointensity persisted in rejected grafts since the label from dead cells is likely taken up by microglia (Berman et al., 2011). In grafts that survived, the signal is lost over time presumably due to the label being diluted during cell division, (Berman et al., 2011). In another study, when SPIO-labeled NPC were injected into the rat brain and subsequently rejected, the hypointensity persisted at the site of injection in both cases, but again no migration was seen (Bernau et al., 2015). This demonstrates that with proper image processing, the appearance of the MR scan could be used to determine the success of a graft. This would comprise an important diagnostic and prognostic tool that could potentially indicate if a NPC transplant had been successful, or if another graft is required. However, to generate that data to inform these algorithms will require more research using disease models in which cell migration away from the site of injection is expected.

The introduction of stem cells into patients raises the possibility of long term complications due to development of tumors from the transplanted cells. A major safety concern with the use of embryonic stem cells is the formation of benign teratomas (Blum et al., 2009). The formation can be reduced by differentiating the cells beyond the pluripotent state in culture prior to transfer; however, differentiation is usually not 100% leaving stem cells and the potential risk. In addition, other factors can influence the formation of tumors from stem cells (Erdo et al., 2003). When NPCs selected from human embryonic stem cells were injected into mouse eyes, teratomas formed in 50% of animals at 8 weeks post-transplant (Arnhold et al., 2004). Tissue devoid of pluripotent stem cells, derived from either fetal or adult cells, may provide a safer mode of cell generation while still maintaining the cells’ potency to differentiate (Pollard et al., 2006; Kosztowski et al., 2009). A majority of the studies in stroke models use fetal or adult cell lines and none report any incidence of tumor formation. In one study, cells implanted into animals were successfully monitored for more than a year (58 weeks) with no report of tumors (Obenaus et al., 2011).

Another obstacle in monitoring exogenously labeled cells is the loss of signal over time due to changes in the cells, particles or both. Reduction in signal has been attributed to several different mechanisms: (1) degradation of the particles over time, (2) dilution of the contrast agent, per cell, during cell proliferation, or (3) release of the particles to endogenous cells upon death of the transplanted NPC. Any of these alone, or in combination, can significantly disrupt plans for prolonged *in vivo* imaging of NPC migration and distribution. By understanding these mechanisms and the vulnerable points in a cell tracking study, they can in part be circumvented.

The degradation of particles is dependent on the chemical and physical properties of the agent used. Particles enter the cells through endocytosis and are then exposed to the acidic environment of the lysosome, leading to degradation of the agent over time. By mimicking the conditions inside of lysosomes (lysosomal conditions: pH 4.5, sodium citrate buffer) it was shown that ferumoxides (Feridex) release free iron after 7 days, and when evaluated in cultured MSCs, the particles aggregated within the lysosomes and began degradation after 5 days (Arbab et al., 2005a). Poly (lactic co-glycolic acid) (PLGA) encapsulated iron oxide nanoparticles persist for a longer period of time under these conditions but undergo a distinct burst and plateau phase; particles degrade by approximately 80% over a 12-week period (Nkansah et al., 2011). There have been no comprehensive studies published that directly examine and compare the dissolution of different particle types in NPC, either *in vitro* or *in vivo*.

Cells can also be transfected with DNA encoding modified forms of the iron storage protein, ferritin, and then transplanted into the brain. The goal would be to increase specificity of the markers and ensure iron persistence over time, while still having the signal inextricably linked to cell viability. When ferritin- or luciferase-transfected (negative control) cells were transplanted into the striatum of healthy rats, similar hypointensities were observed in the brains of these animals (Bernau et al., 2015). This unexpected result is likely due to the fact that injection of either transfected cell type caused hemorrhage at the site of injection. Moreover, the signal loss persisted even when the cells were rejected (observed after withdrawal of an anti-rejection agent), and no migration of the cells was observed this study (Bernau et al., 2015). At the present time, there are no genetically encoded MR reporters that can be transfected into exogenous cells, or endogenous cells, and used for spatiotemporal MR imaging of cell migration *in vivo*.

When NPCs proliferate and differentiate *in vitro* or *in vivo*, particle concentration per cell is diluted with each division. Differentiation can involve asymmetric cell division with different amounts of contrast agent being retained within each daughter cell (Walczak et al., 2007). Labeling cells with Feridex does not seem to influence the ability of NPCs to differentiate (Cohen et al., 2010). However, the rate of differentiation *in vivo* will vary with the tissue environment, i.e., disease model, and the cell type used. In shiverer mouse models of hereditary demyelination, iron oxide loaded cells were not detectable by MR after 6 days presumably due to dilution of the agent with cell division (Walczak et al., 2007). In contrast, similarly labeled cells transferred into models of glioma and stroke could be monitored for weeks without the loss of signal. It is important to note that these anecdotal studies have not been supported, to date, with studies directly examining either cell division rates or the differentiation of transplanted cells in glioma or stroke models. Cell division is typically assessed *ex vivo* using a dye, CFSE, which is diluted with cell division and flow cytometry to assess dye concentrations—such experiments are labor intensive and expensive.

Death of transplanted cells can also confound imaging studies. When implanted NPCs die, particles are released and made available to endogenous cells that can pick up the contrast and shuttled it away from the site of injection, reducing the MRI signal. The mononuclear phagocytic system is the major route of clearance for circulating particles and those found within many organs including the brain, it internalizes and metabolize the free-floating particles (Arami et al., 2015). Excess particles from intraventricular injections are taken up by macrophages and leads to the buildup of iron in macrophage-rich organs including the choroid plexus, liver and spleen (Gorman et al., 2018). Alternatively, the contrast can be taken up by resident microglia to create a false positive signal that persists within the brain. In a variety of different disease models, exogenous NPCs have been shown to primarily differentiate into neurons, astrocytes, and oligodendrocytes and not microglia or macrophages. In a study looking at the injection of dead NPCs, it was demonstrated that particles are found in astrocytes that accumulate at the site of injection and not within cells that migrate (Flexman et al., 2011). This suggests that moving particles may be indicative of live NPC, and stationary particles may have been released by the death of their original host. Nonetheless, there are a number of patterns of iron oxide distribution in the brain following transfer of labeled cells, and these patterns are indicative of the fates and function of both transplanted and endogenous cells. Working out the significance of the hypointensity patterns in MR images may be possible and become useful in guiding the development of cell-based therapies for brain injury and disease.

## The Future of NPCs and MRI

Magnetic resonance imaging has successfully been used to image the migration of endogenous and transplanted NPC in rodents, providing the opportunity to monitor neural stem/progenitor cell fates, function and migration in the brain with applications to the study of brain development and treatment of focal and diffuse disease. However, some questions remain that must be solved before cellular MRI can be used in patients. These largely relate to the impact of the uncertain availability of particles for cell tracking in humans, the basic questions of the mechanism of uptake of particles, the need to transition from small animal models to large animals and humans, and the effect of ever increasing availability of high field clinical MRI scanners.

Current work has shown that the most sensitive labels for imaging are the MPIOs, but these particles have a non-biodegradable polystyrene coating and are unlikely to be approved for use in humans. Other particles such as Feridex have been taken off the market, while ferumoxytol has a black box warning from the FDA. There is a clinical need for biodegradable particles containing large amounts of iron to be developed so that this technique can be applied in human patients. One solution could be iron crystals encapsulated in the clinically approved polymer PLGA; these particles are non-toxic and the size can easily be manipulated during fabrication (Nkansah et al., 2011; Granot et al., 2014).

Magnetic particle imaging (MPI) is an emerging technique for imaging magnetically labeled cells that has recently been applied to stem cell imaging in the brain. MPI directly detects the non-linear magnetization of magnetic particles by measuring the effect of an oscillating magnetic field that is rastered through the sample (Gleich and Weizenecker, 2005). MPI shows promise for direct quantification of the amount of iron (and therefore the number of cells). MSC and hESC-derived neural cells have been injected into the rodent brain and imaged *in vivo* (Bulte et al., 2015; Zheng et al., 2015). Current sensitivity is about 200 cells *in vitro* and 50,000 cells *in vivo* (Bulte et al., 2015; Zheng et al., 2015) and new particles specifically designed for MPI may increase sensitivity. MPI has the advantage that there is no background signal from normal brain tissue. One drawback to this technique is that the MPI image does not contain any anatomical information, so it must be acquired in a scanner that combines MPI and another imaging modality such as MRI or CT to accurately locate the source of the MPI signal. The resolution of the MPI is also quite coarse as it is applied in these papers, on the order of 1 mm (Bulte et al., 2015; Zheng et al., 2015).

One largely unknown factor is the mechanism of particle uptake when cells are labeled *in situ*. An understanding of this process could allow us to design particles that are specific to NPCs (Elvira et al., 2012; Zhong et al., 2015; Zhang et al., 2016). Additionally, there is a trend to develop ‘smart’ contrast agents that specifically cause a signal when they are within a particular, pre-determined, cell type (e.g., Granot and Shapiro, 2011; Gallo et al., 2014); application of this approach to NPC transplantation in the brain would greatly improve these tracking experiments.

In preclinical work, tracking of labeled NPCs has so far been limited to rodent models of disease. Larger animal models, such as cats, dogs, and pigs, should also be used as a precursor to transition to the clinic. The existence of a RMS in humans is currently debated and even proliferation of cells in the adult SVZ is controversial (reviewed in Butti et al., 2014). It is well established, however, that neurogenesis decreased with age (Enwere et al., 2004; Apostolopoulou et al., 2017) and demonstrated via *in situ* labeling to impact migration rates, as well as, NPC total number in the RMS and OB (Shuboni-Mulligan et al., 2018). Further studies are needed to determine if there are treatments that can restart directed proliferation and migration of NPCs in the damaged and diseased brain.

In these imaging studies, the field strength of the MRI system is critical to sensitive detection. Most of the studies described in this review were performed at field strengths above the clinical standard of 3T (at 4.7T, 7T, 9.4T and above). Sensitivity to labeled cells increases with field strength, even in rodent models (Panizzo et al., 2009), and high fields accelerate acquisition of the high-resolution images that are needed for single-cell tracking. However, there may not be a significant gain in sensitivity with increasing field strength (e.g., 7.0T and 11.7T; Shapiro et al., 2005). For translation of NPC tracking to human studies, the importance of high field needs to be determined. Zhu et al. (2006) injected autologous NPCs labeled with SPIOs into patients with traumatic brain injury, and migration of the cells toward the lesion was visible, even with a 3.0T system. In a study of the spine transplanted CD34+ cells labeled with large particles were visible with a 1.0T magnet (Callera and de Melo, 2007). High-field human MRI systems (7.0T) are becoming more common but still present technical challenges related to coil design and energy deposition; however, their use might increase the feasibility and significance of NPC tracking in humans (van der Kolk et al., 2013).

## Conclusion

Enabling MR imaging of NPCs through labeling with iron oxide particles creates an opportunity for examining the migration and homing of NPCs in the healthy and diseased brain. *In situ* labeling with iron oxide particles is achieved by direct injection of particles into the lateral ventricle, these particles are phagocytosed by NPC in the SVZ and carried within the cells as they migrate to the OB. MRI allows for the serial monitoring of single cell migration along the RMS within an individual subject over time. Basic science studies using the technique have revealed the true rate of migration across the RMS *in vivo* and the importance of these migratory cells in olfaction and aging. Pre-clinically, the technique has demonstrated the homing of endogenous NPC to stroke and highlighted the importance of endogenous cells in minimizing the longitudinal negative effects of ischemia, as blocking endogenous migration with Ara-A leads to larger infarcts over time. Labeling methods in rats are well defined and easily replicated between laboratories; particles can be large non-biodegradable particles that are sensitive at the single-cell level or smaller particles that have already received clinical approval. There have been some strides to create particles that are targeted to NPC and would allow for lower dose injections in animal studies. Synthesis of biocompatible PLGA SPIOs are also providing a promising avenue for a clinically viable source of contrast for MRI-based cell tracking. Although there are some remaining challenges to the technique, *in situ* labeling has and will continue to lead to a greater understanding of how these cells function in therapy and the role of NPCs in normalizing the brain.

*In vitro* labeling followed by cellular transplantation into the brain has been used to determine the homing and treatment mechanisms of NPCs in many disease, including stroke, traumatic brain injury, cancer, Parkinson’s disease and multiple sclerosis. The literature of MRI and *in vitro* SPIO labeling is most rich in the fields of stroke and glioma and has lead from basic science experiments into multiple phase I clinical trials. In glioma, exogenous NPC can be derived from patient fibroblast and are genetically altered to synthesize an enzyme that can convert a prodrug into a chemotherapeutic. MRI tracking then allows researchers to watch the migration of the cells into the tumor and give the prodrug at the optimal time to kill as many neighboring tumor cells. This method provides a unique treatment for an aggressive disease, whose standard of care has not altered since the advent of the Stupp protocol in 2009. Methodologically, *in vitro* NPC labeling in rodents has determined the best type of isolated cells for minimal immune response, optimal route of administration, rate of particle degradation, and causes of signal loss. Clearly, the field of regenerative medicine has made great strides in the application of NPC in therapeutics and MRI cell tracking has provided an excellent tool for elevating the research.

The future of NPC tracking using MRI holds many promising avenues to transform both *in situ* and *in vitro* labeling and imaging. Transfection of NPCs with MRI reporter genes is in its infancy and is not yet at a point where it can be adapted for cell tracking studies due to the very low signals compared to signals from cells labeled *in situ* or *in vitro* with iron oxide particles but promises imaging of cellular fates and function. PLGA coated iron oxide nanocrystals provide a possible solution to the current ban on clinically approved cell tracking particles, as they are non-toxic and comprised of a FDA-approved material. Surface manipulation of particles is also being explored to improve NPC uptake *in vitro* and targeting endogenous cells *in situ*. Finally, imaging methods are improving with the growth of human 7.0T MRI and the advent of MPI technology, allowing for higher sensitivity and increased detection of labeled cells. We are at the very early stages of this burgeoning field of research, and the tools being developed hold tremendous promise for improving human health and treating disease.

## Author Contributions

All authors listed have made a substantial, direct and intellectual contribution to the work, and approved it for publication.

## Conflict of Interest Statement

The authors declare that the research was conducted in the absence of any commercial or financial relationships that could be construed as a potential conflict of interest.

## Abbreviations

BrdU: bromodeoxyuridine
GBM: glioblastoma multiforme
heNSC: human embryonic neural stem cell
MCAO: middle cerebral artery occlusion
ML: magnetoliposomes
MPI: magnetic particle imaging
MPIO: micron-sized iron oxide particles
NPC: neural progenitor cell
NSC: neural stem cell
OB: olfactory bulb
PLGA: poly (lactic co-glycolic acid)
RMS: rostral migratory stream
SPIO: superparamagnetic iron oxide nanoparticles
SVZ: subventricular zone
USPIO: ultrasmall superparamagnetic iron oxide particles

## References

[B1] Aboody K. S. Najbauer J. Metz M. Z. D’Apuzzo M. Gutova M. Annala A. J. (2013). Neural stem cell-mediated enzyme/prodrug therapy for glioma: preclinical studies. *Sci. Transl. Med.* 5:184ra59. 10.1126/scitranslmed.3005365 23658244 PMC3864887

[B2] Aboody K. S. Brown A. Rainov K. G. Bower K. A. Liu S. Yang W. (2000). Neural stem cells display extensive tropism for pathology in adult brain: evidence from intracranial gliomas. *Proc. Natl. Acad. Sci. U.S.A.* 97 12846–12851. 10.1073/pnas.97.23.12846 11070094 PMC18852

[B3] Abrous D. N. Koehl M. Moal M. L. (2005). Adult neurogenesis: from precursors to network and physiology. *Physiol. Rev.* 85 523–569. 10.1152/physrev.00055.2003 15788705

[B4] Afridi M. J. Ross A. Liu X. Bennewitz M. F. Shuboni D. D. Shapiro E. M. (2017). Intelligent and automatic *in vivo* detection and quantification of transplanted cells in MRI. *Magn. Reson. Med.* 78 1991–2002. 10.1002/mrm.26571 28019017 PMC5817897

[B5] Akgun I. Unlu M. C. Erdal O. A. Ogut T. Erturk M. Ovali E. (2015). Matrix-induced autologous mesenchymal stem cell implantation versus matrix-induced autologous chondrocyte implantation in the treatment of chondral defects of the knee: a 2-year randomized study. *Arch. Orthop. Trauma Surg.* 135 251–263. 10.1007/s00402-014-2136-z 25548122

[B6] Apostolopoulou M. Kiehl T. R. Winter M. de la Hoz E. C. Boles N. C. Bjorsson C. S. (2017). Non-montonic changes in progenitor cell behavior and gene expression during aging of the adult V-SVZ neural stem cell niche. *Stem Cell Rep.* 9 1931–1947. 10.1016/j.stemcr.2017.10.005 29129683 PMC5785674

[B7] Arami H. Khandhar A. Liggitt D. Krishnan K. M. (2015). *In vivo* delivery, pharmacokinetics, biodistribution and toxicity of iron oxide nanoparticles. *Chem. Soc. Rev.* 44 8576–8607. 10.1039/c5cs00541h 26390044 PMC4648695

[B8] Arbab A. S. Wilson L. B. Ashari P. Jordan E. K. Lewis B. K. Frank J. A. (2005a). A model of lysosomal metabolism of dextran coated superparamagnetic iron oxide (SPIO) nanoparticles: implications for cellular magnetic resonance imaging. *NMR Biomed.* 18 383–389. 16013087 10.1002/nbm.970

[B9] Arbab A. S. Yocum G. T. Rad A. M. Khakoo A. Y. Fellowes V. Read E. J. (2005b). Labeling of cells with ferumoxides–protamine sulfate complexes does not inhibit function or differentiation capacity of hematopoietic or mesenchymal stem cells. *NMR Biomed.* 18 553–559. 10.1002/nbm.991 16229060

[B10] Arnhold S. Klein H. Semkova I. Addicks K. Schraermeyer U. (2004). Neurally selected embryonic stem cells induce tumor formation after long-term survival following engraftment into the subretinal space. *Invest. Opthalmol. Vis. Sci.* 45 4251–4255. 10.1167/iovs.03-1108 15557428

[B11] Arvidsson A. Collin T. Kirik D. Kokaia Z. Lindvall O. (2002). Neuronal replacement from endogenous precursors in the adult brain after stroke. *Nat. Med.* 8 963–970. 10.1038/nm747 12161747

[B12] Ashwal S. Ghosh N. Turenius C. I. Dulcich M. Denham C. M. Tone B. (2014). Reparative effects of neural stem cells in neonatal rats withhypoxic–ischemic injury are not influenced by host sex. *Pediatr. Res.* 75 603–611. 10.1038/pr.2014.7 24463490 PMC4404035

[B13] Aswendt M. Henn N. Michalk S. Schneider G. Steiner M. S. Bissa U. (2015). Novel bimodal iron oxide particles for efficient tracking of human neural stem cells *in vivo*. *Nanomedicine* 10 2499–2512. 10.2217/NNM.15.94 26296195

[B14] Bago J. R. Okolie O. Dumitru R. Ewend M. G. Parker J. S. Werff R. V. (2017). Tumor-homing cytotoxic human unduced neural stem cells for cancer therapy. *Sci. Transl. Med.* 9:eaah6510. 10.1126/scitranslmed.aah6510 28148846 PMC6719790

[B15] Baker E. W. Platt S. R. Lau V. W. Grace H. E. Holmes S. P. Wang L. (2017). Induced pluripotent stem cell-derived neural stem cell theraphy enhances recovery in an ischemic stroke pig model. *Sci. Rep.* 7:10075. 10.1038/s41598-017-10406-x 28855627 PMC5577218

[B16] Barish M. E. Herrmann K. Tang Y. Herculian S. A. Metz M. Aramburo S. (2017). Human neural stem cell biodistribution and predicted tumor coverage by a diffusible therapeutic in a mouse glioma model. *Stem Cells Transl. Med.* 6 1522–1532. 10.1002/sctm.16-0397 28481046 PMC5689763

[B17] Barrow M. Taylor A. Nieves D. J. Bogart L. K. Mandal P. Collins C. M. (2015). Tailoring the surface charge of dextran-based polymer coated SPIONs for modulated stem cell uptake and MRI contrast. *Biomater. Sci.* 3 608–616. 10.1039/c5bm00011d 26222421

[B18] Beckmann N. Falk R. Zurbrügg S. Dawson J. Engelhardt P. (2003). Macrophage infiltration into the rat knee detected by MRI in a model of antigen-induced arthritis. *Magn. Reson. Med.* 49 1047–1055. 10.1002/mrm.10480 12768583

[B19] Ben-Hur T. van Heeswijk R. B. Einstein O. Aharonowiz M. Xue R. Frost E. E. (2007). Serial in vivo MR tracking of magnetically labeled neural spheres transplanted in chronic EAE mice. *Magn. Reson. Med.* 57 164–171. 10.1002/mrm.21116 17191231

[B20] Bennewitz M. F. Tang K. S. Markakis E. A. Shapiro E. M. (2012). Specific chemotaxis of magnetically labeled mesenchymal stem cells: implications for mri of glioma. *Mol. Imaging Biol.* 14 676–687. 10.1007/s11307-012-0553-3 22418788 PMC3388177

[B21] Berman S. C. Galpoththawela C. Gilad A. A. Bulte J. W. M. Walczak P. (2011). Long-term MR cell tracking of neural stem cells grafted in immunocompetent versus immunodeficient mice reveals distinct differences in contrast between live and dead cells. *Magn. Reson. Med.* 65 564–574. 10.1002/mrm.22613 20928883 PMC3031985

[B22] Bernau K. Lewis C. M. Petelinsek A. M. Reagan M. S. Niles D. J. Mattis V. B. (2015). *In Vivo* tracking of human neural progenitor cells in the rat brain using magnetic resonance imaging is not enhanced by ferritin expression. *Cell Transplant.* 25 575–592. 10.3727/096368915X688614 26160767 PMC5052815

[B23] Betarbet R. Zigovz T. Barkay R. Luskin M. (1996). Migration patterns of neonatal subventricular zone progenitor cells transplanted into the neonatal striatum. *Cell Transplant.* 5 165–178. 10.1177/096368979600500207 8689029

[B24] Blum B. Bar-Nur O. Golan-Lev T. Benvenisty N. (2009). The anti-apoptotic gene survivin contributes to teratoma formation by human embryonic stem cells. *Nat. Biotechnol.* 27 281–287. 10.1038/nbt.1527 19252483

[B25] Boese A. C. Le Q.-S. E. Pham D. Hamblin M. H. Lee J.-P. (2018). Neural stem cell therapy for subacute and chronic ischemic stroke. *Stem Cell Res. Ther.* 9:154. 10.1186/s13287-018-0913-2 29895321 PMC5998588

[B26] Brekke C. Williams S. C. Price J. Thorsen F. Modo M. (2007). Cellular multiparametric MRI of neural stem cell therapy in a rat glioma model. *Neuroimage* 37 769–782. 10.1016/j.neuroimage.2007.06.006 17613248

[B27] Brunjes P. C. Armstrong A. M. (1996). Apoptosis in the rostral migratory stream of the developing rat. *Dev. Brain Res.* 92 219–222. 10.1016/0165-3806(96)00006-5 8738129

[B28] Bulte J. W. M. Kraitchman D. L. Mackay A. M. Pittenger M. F. (2004). Chondrogenic differentiation of mesenchymal stem cells is inhibited after magnetic labeling with ferumoxides. *Blood* 104 3410–3413. 10.1182/blood-2004-06-2117 15525839

[B29] Bulte J. W. M. Walczak P. Janowski M. Krishnan K. M. Arami H. Halkola A. (2015). Quantitative “hot spot” imaging of transplanted stem cells using superparamagnetic tracers and magnetic particle imaging (MPI). *Tomography* 1 91–97. 10.18383/j.tom.2015.00172 26740972 PMC4699415

[B30] Butti E. Cusimano M. Bacigaluppi M. Martino G. (2014). Neurogenic and non-neurogenic functions of endogenous neural stem cells. *Front. Neurosci.* 8:92 10.3389/fnins.2014.00092 PMC4010760 24808821

[B31] Callera F. de Melo C. M. (2007). Magnetic resonance tracking of magnetically labeled autologous bone marrow cd34 + cells transplanted into the spinal cord via lumbar puncture technique in patients with chronic spinal cord injury: cd34 + cells’ migration into the injured site. *Stem Cells Dev.* 16 461–466. 10.1089/scd.2007.0083 17610376

[B32] Chaumeil M. M. Gini B. Yang H. Iwanami A. Sukumar S. Ozawa T. (2012). Longitudinal evaluation of MPIO-labeled stem cell biodistribution in glioblastoma using high resolution and contrast-enhanced MR imaging at 14.1Tesla. *Neuro –Oncol.* 14 1050–1061. 10.1093/neuonc/nos126 22670012 PMC3408258

[B33] Christie K. J. Turnley A. M. (2013). Regulation of endogenous neural stem/progenitor cells for neural repair—factors that promote neurogenesis and gliogenesis in the normal and damaged brain. *Front. Cell. Neurosci.* 6:70 10.3389/fncel.2012.00070 PMC3548228 23346046

[B34] Cohen M. E. Muja N. Fainstein N. Bulte J. W. M. Ben-Hur T. (2010). Conserved fate and function of ferumoxides-labeled neural precursor cells *in vitro* and *in vivo*. *J. Neurosci. Res.* 88 936–944. 10.1002/jnr.22277 19885865 PMC3031987

[B35] Daadi M. M. Hu S. Klausner J. Li Z. Sofilos M. Sun G. (2013). Imaging neural stem cell graft-induced structural repair in stroke. *Cell Transplant.* 22 881–892. 10.3727/096368912X656144 23044338 PMC5823270

[B36] Daadi M. M. Li Z. Arac A. Grueter B. A. Sofilos M. Malenka R. C. (2009). Molecular and magnetic resonance imaging of human embryonic stem cell–derived neural stem cell grafts in ischemic rat brain. *Mol. Ther.* 17 1282–1291. 10.1038/mt.2009.104 19436269 PMC2835224

[B37] Daadi M. M. Maag A.-L. Steinberg G. K. (2008). Adherent self-renewable human embryonic stem cell-derived neural stem cell line: functional engraftment in experimental stroke model. *PLoS One* 3:e1644. 10.1371/journal.pone.0001644 18286199 PMC2238795

[B38] Diaz-Coranguez M. Segovia J. Lopez-Ornelas A. Puerta-Guardo H. Ludert J. Chavez B. (2013). Transmigration of neural stem cells across the blood brain barrier induced by glioma cells. *PLoS One* 8:e60655. 10.1371/journal.pone.0060655 23637756 PMC3618035

[B39] Elvira G. Garcia I. Benito M. Gallo J. Desco M. Penades S. (2012). Live imaging of mouse endogenous neural progenitors migrating in response to an induced tumor. *PLoS One* 7:e44466. 10.1371/journal.pone.0044466 22957072 PMC3434138

[B40] Enwere E. Shingo T. Gregg C. Fujikawa H. Ohta S. Weiss S. (2004). Aging results in reduced epidermal growth factor receptor signaling, diminished olfactory neurogenesis, deficits in fine olfactory discrimination. *J. Neurosci.* 24 8354–8365. 10.1523/JNEUROSCI.2751-04.2004 15385618 PMC6729689

[B41] Erdo F. Behrle C. Blunk J. Hoehn M. Xia Y. Fleischmann B. (2003). Host-dependent tumorigenesis of embryonic stem cell transplantation in experimental stroke. *J. Cereb. Blood Flow Metab.* 23 780–785. 10.1097/01.WCB.0000071886.63724.FB 12843782

[B42] Flexman J. A. Cross D. J. Tran L. N. Sasaki T. Kim Y. Minoshima S. (2011). Quantitative analysis of neural stem cell migration and tracer clearance in the rat brain by MRI. *Mol. Imaging Biol.* 13 104–111. 10.1007/s11307-010-0311-3 20440567

[B43] Frank J. A. Zywicke H. Jordan E. K. Mitchell J. Lewis B. K. Miller B. (2002). Magnetic intracellular labeling of mammalian cells by combining (FDA-approved) superparamagnetic iron oxide MR contrast agents and commonly used transfection agents. *Acad. Radiol.* 9 S484–S487. 10.1016/S1076-6332(03)80271-4 12188316

[B44] Gage F. H. (2002). Neurogenesis in the adult brain. *J. Neurosci.* 22 612–613. 10.1523/JNEUROSCI.22-03-00612.2002 11826087 PMC6758482

[B45] Gallo J. Kamaly N. Lavdas I. Stevens E. Nguyen Q.-D. Wylezinska-Arridge M. (2014). CXCR4-Targeted and MMP-responsive iron oxide nanoparticles for enhanced magnetic resonance imaging. *Angew. Chem. Int. Ed.* 53 9550–9554. 10.1002/anie.201405442 25045009 PMC4321346

[B46] García-Verdugo J. M. Doetsch F. Wichterle H. Lim D. A. Alvarez-Buylla A. (1998). Architecture and cell types of the adult subventricular zone: in search of the stem cells. *J. Neurobiol.* 36 234–248. 10.1002/(SICI)1097-4695(199808)36:2<234::AID-NEU10>3.0.CO;2-E 9712307

[B47] Genove G. DeMarco U. Xu H. Goins W. F. Ahrens E. T. (2005). A new transgene reporter for *in vivo* magnetic resonance imaging. *Nat. Med.* 11 450–454. 10.1038/nm1208 15778721

[B48] Glass R. Synowitz M. Kronenberg G. Walzlein J. H. Markovic D. S. Wang L. P. (2005). Glioblastoma-induced attraction of endogenous neural precursor cells is associated with improved survival. *J. Neurosci.* 25 2637–2646. 10.1523/JNEUROSCI.5118-04.2005 15758174 PMC6725181

[B49] Gleich B. Weizenecker J. (2005). Tomographic imaging using the nonlinear response of magnetic particles. *Nature* 435 1214–1217. 15988521 10.1038/nature03808

[B50] Go A. S. Mozaffarian D. Roger V. L. Benjamin E. J. Berry J. D. Blaha M. J. (2014). Heart disease and stroke statistics–2014 update: a report from the american heart association. *Circulation* 129 e28–e292. 10.1038/nature03808 24352519 PMC5408159

[B51] Gonzalez-Perez O. (2012). Neural stem cells in the adult human brain. *Biol. Biomed. Rep.* 2 59–69. 10.1161/01.cir.0000441139.02102.80 23181200 PMC3505091

[B52] Gorman A. W. Deh K. M. Schwiedrzik C. M. White J. R. Groman E. V. Fisher C. A. (2018). Brain iron distribution after multiple doses of ultra-small supraparamagnetic iron oxide particles in rats. *Comp. Med.* 68 139–147. 29663939 PMC5897970

[B53] Granot D. Nkansah M. K. Bennewitz M. F. Tang K. S. Markakis E. A. Shapiro E. M. (2014). Clinically viable magnetic poly(lactide-co-glycolide) particles for MRI-based cell tracking. *Magn. Reson. Med.* 71 1238–1250. 10.1002/mrm.24741 23568825 PMC4336220

[B54] Granot D. Scheinost D. Markakis E. A. Papademetris X. Shapiro E. M. (2011). Serial monitoring of endogenous neuroblast migration by cellular MRI. *Neuroimage* 57 817–824. 10.1016/j.neuroimage.2011.04.063 21571076 PMC3129484

[B55] Granot D. Shapiro E. M. (2011). Release activation of iron oxide nanoparticles: (reaction) A novel environmentally sensitive MRI paradigm. *Magn. Reson. Med.* 65 1253–1259. 10.1002/mrm.22839 21360745 PMC3119922

[B56] Granot D. Shapiro E. M. (2014). Accumulation of micron sized iron oxide particles in endothelin-1 induced focal cortical ischemia in rats is independent of cell migration. *Magn. Reson. Med.* 71 1568–1574. 10.1002/mrm.24788 23661604

[B57] Guglielmetti C. Praet J. Rangarajan J. R. Vreys R. De Vocht N. Maes F. (2014). Multimodal imaging of subventricular zone neural stem/progenitor cells in the cuprizone mouse model reveals increased neurogenic potential for the olfactory bulb pathway, but no contribution to remyelination of the corpus callosum. *Neuroimage* 86 99–110. 10.1016/j.neuroimage.2013.07.080 23933305

[B58] Gutova M. Frank J. A. D’Apuzzo M. Khankaldyyan V. Gilchrist M. M. Annala A. J. (2013). Magnetic resonance imaging tracking of ferumoxytol-labeled human neural stem cells: studies leading to clinical use. *Stem Cells Transl. Med.* 2 766–775. 10.5966/sctm.2013-0049 24014682 PMC3785261

[B59] Guzman R. Bliss T. De Los Angeles A. Moseley M. Palmer T. Steinberg G. (2008). Neural progenitor cells transplanted into the uninjured brain undergo targeted migration after stroke onset. *J. Neurosci. Res.* 86 873–882. 10.1002/jnr.21542 17975825

[B60] Guzman R. Uchida N. Bliss T. M. He D. Christopherson K. K. Stellwagen D. (2007). Long-term monitoring of transplanted human neural stem cells in developmental and pathological contexts with MRI. *Proc. Natl. Acad. Sci. U.S.A.* 104 10211–10216. 10.1073/pnas.0608519104 17553967 PMC1891235

[B61] Haacke E. M. Liu S. Buch S. Zheng W. Wu D. Ye Y. (2015). Quantitative susceptibility mapping: current status and future directions. *Magn. Reson. Imaging* 33 1–25. 10.1016/j.mri.2014.09.004 25267705

[B62] Hare J. M. Fishman J. E. Gerstenblith G. DiFede Velazquez D. L. Zambrano J. P. Suncion V. Y. (2012). Comparison of allogeneic vs autologous bone marrow–derived mesenchymal stem cells delivered by transendocardial injection in patients with ischemic cardiomyopathy: the poseidon randomized trial. *JAMA* 308 2369–2379. 10.1001/jama.2012.25321 23117550 PMC4762261

[B63] Harris V. K. Stark J. Vyshkina T. Blackshear L. Joo G. Stefanova V. (2018). Phase I trial of intrathecal mesenchymal stem cell-derived neural progenitors in progressive multiple sclerosis. *eBioMed.* 29 23–30. 10.1016/j.ebiom.2018.02.002 29449193 PMC5925446

[B64] Heyn C. Ronald J. A. Mackenzie L. T. MacDonald I. C. Chambers A. F. Rutt B. K. (2006a). In vivo magnetic resonance imaging of single cells in mouse brain with optical validation. *Magn. Reson. Med.* 55 23–29. 10.1002/mrm.20747 16342157

[B65] Heyn C. Ronald J. A. Ramadan S. S. Snir J. A. Barry A. M. MacKenzie L. T. (2006b). *In vivo* MRI of cancer cell fate at the single-cell level in a mouse model of breast cancer metastasis to the brain. *Magn. Reson. Med.* 56 1001–1010. 17029229 10.1002/mrm.21029

[B66] Hoehn M. Küstermann E. Blunk J. Wiedermann D. Trapp T. Wecker S. (2002). Monitoring of implanted stem cell migration *in vivo*: a highly resolved *in vivo* magnetic resonance imaging investigation of experimental stroke in rat. *Proc. Natl. Acad. Sci. U.S.A.* 99 16267–16272. 10.1073/pnas.242435499 12444255 PMC138600

[B67] Hong W. He Q. Fan S. Carl M. Shao H. Chen J. (2017). Imaging and quantification of iron-oxide nanoparticles (IONP) using MP-RAGE and UTE based sequences. *Magn. Reson. Med.* 78 226–232. 10.1002/mrm.26371 27495266 PMC5503673

[B68] Huang L. Wong S. Snyder E. Y. Hamblin M. H. Lee J.-P. (2014). Human neural stem cells rapidly ameliorate symptomatic inflammation in early-stage ischemic-reperfusion cerebral injury. *Stem Cell Res. Ther.* 5:129. 10.1186/scrt519 25418536 PMC4445985

[B69] Ihrie R. A. Álvarez-Buylla A. (2011). Lake-front property: a unique germinal niche by the lateral ventricles of the adult brain. *Neuron* 70 674–686. 10.1016/j.neuron.2011.05.004 21609824 PMC3346178

[B70] Iordanova B. Ahrens E. T. (2012). *In vivo* magnetic resonance imaging of ferritin-based reporter visualizes native neuroblast migration. *Neuroimage* 59 1004–1012. 10.1016/j.neuroimage.2011.08.068 21939774 PMC3230706

[B71] Iordanova B. Goins W. F. Clawson D. S. Hitchens T. K. Ahrens E. T. (2013). Quantification of HSV-1-mediated expression of the ferritin MRI reporter in the mouse brain. *Gene Ther.* 20 589–596. 10.1038/gt.2012.70 22996196 PMC5796774

[B72] Iordanova B. Robison C. S. Ahrens E. T. (2010). Design and characterization of a chimeric ferritin with enhanced iron loading and transverse NMR relaxation rate. *J. Biol. Inorg. Chem.* 15 957–965. 10.1007/s00775-010-0657-7 20401622 PMC2936821

[B73] Jiang Q. Zhang Z. G. Ding G. L. Zhang L. Ewing J. R. Wang L. (2005). Investigation of neural progenitor cell induced angiogenesis after embolic stroke in rat using MRI. *Neuroimage* 28 698–707. 10.1016/j.neuroimage.2005.06.063 16112879

[B74] Kalladka D. Sinden J. Pollock K. Haig C. McLean J. Smith W. (2016). Human neural stem cells in patients with chronic ischaemic stroke (PISCES): a phase 1, first-in-man study. *Lancet* 388 787–796. 10.1016/S0140-6736(16)30513-X 27497862

[B75] Kalladka D. Sinden J. Pollock K. McLean J. Dunn L. Santosh C. (2013). Pilot investigation of stem cells in stroke [pisces]. *Cerebrovasc. Dis.* 35 (Suppl. 3):551.

[B76] Kassis I. Vaknin-Dembinsky A. Bulte J. Karussis D. (2010). Effects of supermagnetic iron oxide labeling on the major functional properties of human mesenchymal stem cells from multiple sclerosis patients. *Int. J. Stem Cells* 3 144–153. 10.15283/ijsc.2010.3.2.144 24855552 PMC4021808

[B77] Kleinschnitz C. Bendszus M. Frank M. Solymosi L. Toyka K. V. Stoll G. (2003). *In Vivo* monitoring of macrophage infiltration in experimental ischemic brain lesions by magnetic resonance imaging. *J. Cereb. Blood Flow Metab.* 23 1356–1361. 10.1097/01.WCB.0000090505.76664.DB 14600443

[B78] Kim S. J. Lewis B. Steiner M-S. Bissa U. V. Dose C. Frank J. A. (2016). Superparamagnetic iron oxide nanoparticles for direct labeling of stem cells and *in vivo* MRI tracking. *Contrast Media Mol. Imaging* 11 55–64. 10.1002/cmmi.1658 26234504 PMC4729653

[B79] Kochanek K. D. Xu J. Murphy S. L. Mini-o A. M. Kung H.-C. (2011). National Vital Statistics Reports. Hyattsville, MD: National Center for Health Statistics.

[B80] Kode J. A. Mukherjee S. Joglekar M. V. Hardikar A. A. (2009). Mesenchymal stem cells: immunobiology and role in immunomodulation and tissue regeneration. *Cytotherapy* 11 377–391. 10.1080/14653240903080367 19568970

[B81] Kokovay E. Goderie S. Wang Y. Lotz S. Lin G. Sun Y. (2010). Adult SVZ Lineage cells home to and leave the vascular niche via differential responses to SDF1/CXCR4 signaling. *Cell Stem Cell* 7 163–173. 10.1016/j.stem.2010.05.019 20682445 PMC2916873

[B82] Kosztowski T. Zaidi H. A. Quiñones-Hinojosa A. (2009). Applications of neural and mesenchymal stem cells in the treatment of gliomas. *Expert Rev. Anticancer Ther.* 9 597–612. 10.1586/era.09.22 19445577 PMC2705652

[B83] Kwiatkowska A. Nandhu M. Behera P. Chiocca E. Viapiano M. (2013). Strategies in gene therapy for glioblastoma. *Cancers* 5 1271–1305. 10.3390/cancers5041271 24202446 PMC3875940

[B84] Lamm O. Ganz J. Melamed E. Offen D. (2014). Harnessing neurogenesis for the possible treatment of Parkinson’s disease. *J. Comp. Neurol.* 522 2817–2830. 10.1002/cne.23607 24723264

[B85] Lepore A. C. Walczak P. Rao M. S. Fischer I. Bulte J. W. M. (2006). MR imaging of lineage-restricted neural precursors following transplantation into the adult spinal cord. *Exp. Neurol.* 201 49–59. 10.1016/j.expneurol.2006.03.032 16764862

[B86] Li L. Jiang Q. Ding G. Zhang L. Zhang Z. G. Li Q. (2010). Effects of administration route on migration and distribution of neural progenitor cells transplanted into rats with focal cerebral ischemia, an MRI study. *J. Cereb. Blood Flow Metab.* 30 653–662. 10.1038/jcbfm.2009.238 19888287 PMC2844252

[B87] Li L. Jiang Q. Zhang L. Ding G. Wang L. Zhang R. (2006). Ischemic cerebral tissue response to subventricular zone cell transplantation measured by iterative self-organizing data analysis technique algorithm. *J. Cereb. Blood Flow Metab.* 26 1366–1377. 10.1038/sj.jcbfm.9600288 16511501

[B88] Lin G. He X. Liang F. Guo Y. Sunnassee G. Cao X. (2018). Transplanted human neural precursor cells integrate into the host neural circuit and ameliorate neurological deficits in a mouse model of traumatic brain injury. *Neurosci. Lett.* 674 11–17. 10.1016/j.neulet.2018.02.064 29501684

[B89] Lu C. Hsiao J. Liu H. Wu C. (2017). Characterization of an iron oxide nanoparticle labelling and MRI-based protocol for inducing human mesenchymal stem cells into neural-like cells. *Sci. Rep.* 7:3587. 10.1038/s41598-017-03863-x 28620162 PMC5472606

[B90] Magnitsky S. Pickup S. Garwood M. Idiyatullin D. (2018). Imaging of a high concentration of iron labeled cells with positive contrast in a rat knee. *Magn. Reson. Med.* 10.1002/mrm.27520 [Epub ahead of print]. 30242896 PMC6347481

[B91] Magnitsky S. Zhang J. Idiyatullin D. Mohan G. Garwood M. Lane N. E. (2017). Positive contrast from cells labeled with iron oxide nanoparticles: quantitation of imaging data. *Magn. Reson. Med.* 78 1900–1910. 10.1002/mrm.26585 28097749 PMC5513790

[B92] Mason C. Dunnill P. (2008). A brief definition of regenerative medicine. *Regen. Med.* 3 1–5. 10.2217/17460751.3.1.1 18154457

[B93] Mills P. H. Hitchens T. K. Foley L. M. Link T. Ye Q. Weiss C. R. (2011). Automated detection and characterization of SPIO-labeled cells and capsules using magnetic field perturbations. *Magn. Reson. Med.* 67 278–289. 10.1002/mrm.22998 21656554 PMC3170691

[B94] Mills P. H. Wu Y. Ho C. Ahrens E. T. (2008). Sensitive and automated detection of iron-oxide-labeled cells using phase image cross-correlation analysis. *Magn. Reson. Imaging* 26 618–628. 10.1016/j.mri.2008.01.007 18450402 PMC3200563

[B95] Modo M. Beech J. S. Meade T. J. Williams S. C. R. Price J. (2009). A chronic 1 year assessment of MRI contrast agent-labelled neural stem cell transplants in stroke. *Neuroimage* 47 T133–T142. 10.1016/j.neuroimage.2008.06.017 18634886 PMC4145694

[B96] Modo M. Mellodew K. Cash D. Fraser S. E. Meade T. J. Price J. (2004). Mapping transplanted stem cell mgration after a stroke: a serial, *in vivo* magnetic resonance imaging study. *Neuroimage* 21 311–317. 10.1016/j.neuroimage.2003.08.030 14741669

[B97] Mori Y. Chen T. Fujisawa T. Kobashi S. Ohno K. Yoshida S. (2014). From cartoon to real time MRI: *in vivo* monitoring of phagocytes migration in mouse brain. *Sci. Rep.* 4:6997. 10.1038/srep06997 25385430 PMC4227027

[B98] Morshed R. A. Gutova M. Juliano J. Barish M. E. Hawkins-Daarud A. Oganesyan D. (2015). Analysis of glioblastoma tumor coverage by oncolytic virus-loaded neural stem cells using MRI-based tracking and histological reconstruction. *Cancer Gene Ther.* 22 55–61. 10.1038/cgt.2014.72 25525033 PMC4293243

[B99] Muldoon L. L. Tratnyek P. G. Jacobs P. M. Doolittle N. D. Christoforidis G. A. Frank J. A. (2006). Imaging and nanomedicine for diagnosis and therapy in the central nervous system: report of the eleventh annual blood-brain barrier disruption consortium meeting. *Am. J. Neuroradiol.* 27 715–721. 16552023 PMC7976977

[B100] Mundim M. V. Zamproni L. N. Pinto A. A. S. Galindo L. T. Xavier A. M. Glezer I. (2019). A new function for prokineticin 2: recruitment of SVZ-derived neuroblasts to the injured cortex in a mouse model of traumatic brain injury. *Mol. Cell. Neurosci.* 94 1–10. 10.1016/j.mcn.2018.10.004 30391355

[B101] Mutukula N. Elkabetz Y. (2017). “Neural killer” cells: autologous cytotoxic neural stem cells for fighting glioma. *Cell Stem Cell* 20 426–428. 10.1016/j.stem.2017.03.019 28388425

[B102] Nam S. C. Kim Y. Dryanovski D. Walker A. Goings G. Woolfrey K. (2007). Dynamic features of postnatal subventricular zone cell motility: a two-photon time-lapse study. *J. Comp. Neurol.* 505 190–208. 10.1002/cne.21473 17853439

[B103] Neuwelt E. A. Weissleder R. Nilaver G. Kroll R. A. Roman-Goldstein S. Szumowski J. (1994). Delivery of virus-sized iron oxide particles to rodent CNS neurons. *Neurosurgery* 34 777–784. 8008188 10.1227/00006123-199404000-00048

[B104] Nieman B. J. Shyu J. Y. Rodriguez J. J. Garcia A. D. Joyner A. L. Turnbull D. H. (2010). *In vivo* MRI of neural cell migration dynamics in the mouse brain. *Neuroimage* 50 456–464. 10.1016/j.neuroimage.2009.12.107 20053381 PMC2899477

[B105] Nkansah M. K. Thakral D. Shapiro E. M. (2011). Magnetic poly(lactide-co-glycolide) and cellulose particles for MRI-based cell tracking. *Magn. Reson. Med.* 65 1776–1785. 10.1002/mrm.22765 21404328 PMC3097259

[B106] Nucci L. P. Silva H. R. Giampaoli V. Mamani J. B. Nucci M. P. Gamarra L. F. (2015). Stem cells labeled with supraparamagnetic iron oxide nanoparticles in a preclinical model of cerebral ischemia: a systematic review with meta-analysis. *Stem Cell Res. Ther.* 6:27. 10.1186/s13287-015-0015-3 25889904 PMC4425914

[B107] Obenaus A. Dilmac N. Tone B. Tian H. R. Hartman R. Digicaylioglu M. (2011). Long-term magnetic resonance imaging of stem cells in neonatal ischemic injury. *Ann. Neurol.* 69 282–291. 10.1002/ana.22168 21387373 PMC3069664

[B108] Ogura K. Mizowaki T. Arakawa Y. Ogura M. Sakanaka K. Miyamoto S. (2013). Initial and cumulative recurrence patterns of glioblastoma after temozolomide-based chemoradiotherapy and salvage treatment: a retrospective cohort study in a single institution. *Radiat. Oncol.* 8:97. 10.1186/1748-717X-8-97 24499582 PMC3643853

[B109] Panizzo R. A. Kyrtatos P. G. Price A. N. Gadian D. G. Ferretti P. Lythgoe M. F. (2009). *In vivo* magnetic resonance imaging of endogenous neuroblasts labelled with a ferumoxide–polycation complex. *Neuroimage* 44 1239–1246. 10.1016/j.neuroimage.2008.10.062 19059485

[B110] Park J. K. Hodges T. Arko L. Shen M. Dello Iacono D. McNabb A. (2010). Scale to predict survival after surgery for recurrent glioblastoma multiforme. *J. Clin. Oncol.* 28 3838–3843. 10.1200/JCO.2010.30.0582 20644085 PMC2940401

[B111] Peng H. Huang Y. Rose J. Erichsen D. Herek S. Fujii N. (2004). Stromal cell-derived factor 1-mediated CXCR4 signaling in rat and human cortical neural progenitor cells. *J. Neurosci. Res.* 76 35–50. 10.1002/jnr.20045 15048928

[B112] Pollard S. M. Conti L. Sun Y. Goffredo D. Smith A. (2006). Adherent Neural Stem (NS) cells from fetal and adult forebrain. *Cereb. Cortex* 16 i112–i120. 10.1093/cercor/bhj167 16766697

[B113] Pollock K. Stroemer P. Patel S. Stevanato L. Hope A. Miljan E. (2006). A conditionally immortal clonal stem cell line from human cortical neuroepithelium for the treatment of ischemic stroke. *Exp. Neurol.* 199 143–155. 10.1016/j.expneurol.2005.12.011 16464451

[B114] Pothayee N. Cummings D. M. Schoenfeld T. J. Dodd S. Cameron H. A. Belluscio L. (2017). Magnetic resonance imaging of odorant activity-dependent migration of neural precursor cells and olfactory bulb growth. *Neuroimage* 158 232–241. 10.1016/j.neuroimage.2017.06.060 28669915 PMC5614830

[B115] Qiao L. Huang F. Zhao M. Xie J. Shi J. Wang J. (2014). A two-year follow-up study of cotransplantation with neural stem/progenitor cells and mesenchymal stromal cells in ischemic stroke patients. *Cell Transplant.* 23 65–72. 10.3727/096368914X684961 25333752

[B116] Ramos-Gomez M. Martinez-Serrano A. (2016). Tracking of iron-labeled human neural stem cells by magnetic resonance imaging in cell replacement therapy for Parkinson’s Disease. *Neural Regen. Res.* 11 49–52. 10.4103/1673-5374.169628 26981077 PMC4774222

[B117] Reaux-Le Goazigo A. Van Steenwinckel J. Rostene W. Parsadaniantz S. M. (2013). Current status of chemokines in the adult CNS. *Prog. Neurobiol.* 104 67–92. 10.1016/j.pneurobio.2013.02.001 23454481

[B118] Reekmans K. P. Praet J. De Vocht N. Tambuyzer B. R. Bergwerf I. Daans J. (2011). Clinical potential of intravenous neural stem cell delivery for treatment of neuroinflammatory disease in mice? *Cell Transplant.* 20 851–869. 10.3727/096368910X543411 21092405

[B119] Ring H. L. Zhang J. Klein N. D. Eberly L. E. Haynes C. L. Garwood M. (2018). Establishing the overlap of IONP quantification with echo and echoless MR relaxation mapping. *Magn. Reson. Med.* 79 1420–1428. 10.1002/mrm.26800 28653344 PMC5743777

[B120] Rogall R. Rabenstein M. Vay S. Bach A. Pikhovych A. Baermann J. (2018). Bioluminescence imaging visualizes osteopontin-induced neurogenesis and neuroblast migration in the mouse brain after stroke. *Stem Cell Res. Ther.* 9:182. 10.1186/s13287-018-0927-9 29973246 PMC6032781

[B121] Rogelius N. Ericson C. Lundberg C. (2005). *In vivo* labeling of neuroblasts in the subventricular zone of rats. *J. Neurosci. Methods* 142 285–293. 10.1016/j.jneumeth.2004.09.008 15698668

[B122] Seaberg R. M. van der Kooy D. (2003). Stem and progenitor cells: the premature desertion of rigorous definitions. *Trends Neurosci.* 26 125–131. 10.1016/S0166-2236(03)00031-6 12591214

[B123] Shapiro E. M. (2015). Biodegradable, polymer encapsulated, metal oxide particles for MRI-based cell tracking. *Magn. Reson. Med.* 73 376–389. 10.1002/mrm.25263 24753150 PMC4336226

[B124] Shapiro E. M. Gonzalez-Perez O. Manuel García-Verdugo J. Alvarez-Buylla A. Koretsky A. P. (2006a). Magnetic resonance imaging of the migration of neuronal precursors generated in the adult rodent brain. *Neuroimage* 32 1150–1157. 16814567 10.1016/j.neuroimage.2006.04.219 PMC4035244

[B125] Shapiro E. M. Sharer K. Skrtic S. Koretsky A. P. (2006b). In vivo detection of single cells by MRI. *Magn. Reson. Med.* 55 242–249. 16416426 10.1002/mrm.20718

[B126] Shapiro E. M. Skrtic S. Koretsky A. P. (2005). Sizing it up: cellular MRI using micron-sized iron oxide particles. *Magn. Reson. Med.* 53 329–338. 10.1002/mrm.20342 15678543

[B127] Shapiro E. M. Skrtic S. Sharer K. Hill J. M. Dunbar C. E. Koretsky A. P. (2004). MRI detection of single particles for cellular imaging. *PNAS* 101 10901–10906. 10.1073/pnas.0403918101 15256592 PMC503717

[B128] Shuboni-Mulligan D. D. Chakravarty S. Mallett C. L. Wolf A. M. Forton S. Shapiro E. M. (2018). Age-dependent visualization of neural progenitor cells within the rostral migratory stream via MRI and endogenously labeled micron-sized iron oxide particles. *bioRxiv.* 10.1101/429787

[B129] Song C. Wang J. Mo C. Mu S. Jiang X. Li X. (2015). Use of ferritin expression, regulated by neural cell-specific promoters in human adipose tissue-derived mesenchymal stem cells, to monitor differentiation with magnetic resonance imaging *in vitro*. *PLoS One* 10:e0132480. 10.1371/journal.pone.0132480 26176961 PMC4503445

[B130] Song M. Kim Y. Kim Y. Ryu S. Song I. Kim S. U. (2009). MRI tracking of intravenously transplanted human neural stem cells in rat focal ischemia model. *Neurosci. Res.* 64 235–239. 10.1016/j.neures.2009.03.006 19428705

[B131] Song M. Moon W. K. Kim Y. Lim D. Song I. Yoon B. (2007). Labeling efficiency of supraparamagnetic iron oxide nanoparticles to human neural stem cells: comparison of ferumoxides, monocrystalline iron oxide, cross-linked iron oxide (clio)-nh2 and tat-Clio. *Korean J. Radiol.* 8 365–371. 10.3348/kjr.2007.8.5.365 17923778 PMC2626816

[B132] Sumner J. P. Conroy R. Shapiro E. Moreland J. Koretsky A. P. (2007). Delivery of fluorescent probes using iron oxide particels as carriers enables in vivo labeling of migrating neural progenitors for MRI and optical imaging. *J. Biomed. Opt.* 12:0515504. 17994868 10.1117/1.2800294 PMC3529473

[B133] Sumner J. P. Shapiro E. M. Maric D. Conroy R. Koretsky A. P. (2009). *In vivo* labeling of adult neural progenitors for MRI with micron sized particles of iron oxide: quantification of labeled cell phenotype. *Neuroimage* 44 671–678. 10.1016/j.neuroimage.2008.07.050 18722534 PMC2967480

[B134] Suzuki S. O. Goldman J. E. (2003). Multiple cell populations in the early postnatal subventricular zone take distinct migratory pathways: a dynamic study of glial and neuronal progenitor migration. *J. Neurosci.* 23 4240–4250. 10.1523/JNEUROSCI.23-10-04240.2003 12764112 PMC6741090

[B135] Tai J. H. Foster P. Rosales A. Feng B. Hasilo C. Martinez V. (2006). Imaging islets labeled with magnetic nanoparticles at 1.5 tesla. *Diabetes* 55 2931–2938. 10.2337/db06-0393 17065328

[B136] Thu M. S. Najbauer J. Kendall S. E. Harutyunyan I. Sangalang N. Gutova M. (2009). Iron labeling and pre-clinical MRI visualization of therapeutic human neural stem cells in a murine glioma model. *PLoS One* 4:e7218. 10.1371/journal.pone.0007218 19787043 PMC2746284

[B137] Towner R. A. Smith N. Doblas S. He T. (2011). “Assessment of rodent glioma models using magnetic resonance imaging techniques,” in *Advances in the Biology, Imaging and Therapies for Glioblastoma*, ed. Chen C. C. (Rijeka: InTech), 251–272.

[B138] Tsankova N. M. Canoll P. (2014). Advances in genetic and epigenetic analyses of gliomas: a neuropathological perspective. *J. Neurooncol.* 119 481–490. 10.1007/s11060-014-1499-x 24962200

[B139] van der Kolk A. G. Hendrikse J. Zwanenburg J. J. M. Visser F. Luijten P. R. (2013). Clinical applications of 7 T MRI in the brain. *Eur. J. Radiol.* 82 708–718. 10.1016/j.ejrad.2011.07.007 21937178

[B140] Vande Velde G. Raman Rangarajan J. Vreys R. Guglielmetti C. Dresselaers T. Verhoye M. (2012). Quantitative evaluation of MRI-based tracking of ferritin-labeled endogenous neural stem cell progeny in rodent brain. *Neuroimage* 62 367–380. 10.1016/j.neuroimage.2012.04.040 22677164

[B141] Vande Velde G. Rangarajan J. R. Toelen J. Dresselaers T. Ibrahimi A. Krylychkina O. (2011). Evaluation of the specificity and sensitivity of ferritin as an MRI reporter gene in the mouse brain using lentiviral and adeno-associated viral vectors. *Gene Ther.* 18 594–605. 10.1038/gt.2011.2 21346786

[B142] Ventura R. E. Goldman J. E. (2007). Dorsal radial glia generate olfactory bulb interneurons in the postnatal murine brain. *J. Neurosci.* 27 4297–4302. 10.1523/JNEUROSCI.0399-07.2007 17442813 PMC6672317

[B143] Vreys R. Soenen S. J. H. De Cuyper M. Van der Linden A. (2011). Background migration of USPIO/MLs is a major drawback for in situ labeling of endogenous neural progenitor cells. *Contrast Media Mol. Imaging* 6 1–6. 10.1002/cmmi.390 20648643

[B144] Vreys R. Velde G. V. Krylychkina O. Vellema M. Verhoye M. Timmermans J.-P. (2010). MRI visualization of endogenous neural progenitor cell migration along the RMS in the adult mouse brain: validation of various MPIO labeling strategies. *Neuroimage* 49 2094–2103. 10.1016/j.neuroimage.2009.10.034 19850132

[B145] Walczak P. Kedziorek D. A. Gilad A. A. Barnett B. P. Bulte J. W. M. (2007). Applicability and limitations of MR tracking of neural stem cells with asymmetric cell division and rapid turnover: the case of the Shiverer dysmyelinated mouse brain. *Magn. Reson. Med.* 58 261–269. 10.1002/mrm.21280 17654572

[B146] Wang Y. Liu T. (2015). Quantitative susceptibility mapping (QSM): decoding MRI data for a tissue magnetic biomarker. *Magn. Reson. Med.* 73 82–101. 10.1002/mrm.25358 25044035 PMC4297605

[B147] Wang Y. Rudd A. G. Wolfe C. D. A. (2013). Age and ethnic disparities in incidence of stroke over time: the South London stroke register. *Stroke* 44 3298–3304. 10.1161/STROKEAHA.113.002604 24114452

[B148] Wen X. Wang Y. Zhang F. Zhang X. Lu L. Shuai X. (2014). *In vivo* monitoring neural stem cells after transplantation in acute cerebral infarction with dual-modal MR imaging and optical imaging. *Biomaterial* 35 4627–4635. 10.1016/j.biomaterials.2014.02.042 24631249

[B149] Willing A. E. Shahaduzzaman M. (2013). “Delivery routes for cell therapy in stroke,” in *Cell-Based Therapies in Stroke*, eds Jolkkonen J. Walczak P. (Vienna: Springer), 15–28. 10.1007/978-3-7091-1175-8_2

[B150] Winner B. Cooper-Kuhn C. M. Aigner R. Winkler J. Kuhn H. G. (2002). Long-term survival and cell death of newly generated neurons in the adult rat olfactory bulb. *Eur. J. Neurosci.* 16 1681–1689. 10.1046/j.1460-9568.2002.02238.x 12431220

[B151] Xie D. Qiu B. Walczak P. Li X. Ruiz-Cabello J. Minoshima S. (2010). Optimization of magnetosonoporation for stem cell labeling. *NMR Biomed.* 23 480–484. 10.1002/nbm.1485 20213856

[B152] Yang J. Liu J. Niu G. Chan K. C. Wang R. Liu Y. (2009). *In vivo* MRI of endogenous stem/progenitor cell migration from subventricular zone in normal and injured developing brains. *Neuroimage* 48 319–328. 10.1016/j.neuroimage.2009.06.075 19591946

[B153] Zhang F. Duan X. Lu L. Zhang X. Chen M. Mao J. (2017). *In vivo* long-term tracking of neural stem cells transplanted into an acute ischemic stroke model with reporter gene-based bimodal MR and optical imaging. *Cell Transplant.* 26 1648–1662. 10.1177/0963689717722560 29251112 PMC5753979

[B154] Zhang F. Duan X. Lu L. Zhang X. Zhong X. Mao J. (2016). *In vivo* targeting MR Imaging of endogenous neural stem cells in Ischemic Stroke. *Molecules* 21:E1143. 10.3390/molecules21091143 27589699 PMC6273863

[B155] Zhang Z. Jiang Q. Jiang F. Ding G. Zhang R. Wang L. (2004). *In vivo* magnetic resonance imaging tracks adult neural progenitor cell targeting of brain tumor. *Neuroimage* 23 281–287. 10.1016/j.neuroimage.2004.05.019 15325375

[B156] Zhang Z. G. Jiang Q. Zhang R. Zhang L. Wang L. Zhang L. (2003). Magnetic resonance imaging and neurosphere therapy of stroke in rat. *Ann. Neurol.* 53 259–263. 10.1002/ana.10467 12557295

[B157] Zheng B. Vazin T. Goodwill P. W. Conway A. Verma A. Ulku Saritas E. (2015). Magnetic particle imaging tracks the long-term fate of *in vivo* neural cell implants with high image contrast. *Sci. Rep.* 5:14055. 10.1038/srep14055 26358296 PMC4566119

[B158] Zhong X. Zhang F. Yang M. Wen X. Zhang X. Duan X. (2015). In vivo targeted magnetic resonance imaging of endogenous neural stem cells in the adult rodent brain. *Biomed. Res. Int.* 2015:131054. 10.1155/2015/131054 26583085 PMC4637027

[B159] Zhu J. Zhou L. XingWu F. (2006). Tracking neural stem cells in patients with brain trauma. *N. Engl. J. Med.* 355 2376–2378. 10.1056/NEJMc055304 17135597

